# Proteomic Research on the Antitumor Properties of Medicinal Mushrooms

**DOI:** 10.3390/molecules26216708

**Published:** 2021-11-05

**Authors:** Boris Jakopovic, Nada Oršolić, Ivan Jakopovich

**Affiliations:** 1 Dr Myko San–Health from Mushrooms Co., Miramarska Cesta 109, HR-10000 Zagreb, Croatia; ivan.jakopovic@mykosan.com; 2 Division of Animal Physiology, Faculty of Science, University of Zagreb, Rooseveltov trg 6, HR-10000 Zagreb, Croatia; nada.orsolic@biol.pmf.hr

**Keywords:** cancer, medicinal mushrooms, proteomics, bioinformatics

## Abstract

Medicinal mushrooms are increasingly being recognized as an important therapeutic modality in complementary oncology. Until now, more than 800 mushroom species have been known to possess significant pharmacological properties, of which antitumor and immunomodulatory properties have been the most researched. Besides a number of medicinal mushroom preparations being used as dietary supplements and nutraceuticals, several isolates from mushrooms have been used as official antitumor drugs in clinical settings for several decades. Various proteomic approaches allow for the identification of a large number of differentially regulated proteins serendipitously, thereby providing an important platform for a discovery of new potential therapeutic targets and approaches as well as biomarkers of malignant disease. This review is focused on the current state of proteomic research into antitumor mechanisms of some of the most researched medicinal mushroom species, including *Phellinus linteus*, *Ganoderma lucidum*, *Auricularia auricula*, *Agrocybe aegerita*, *Grifola frondosa*, and *Lentinus edodes*, as whole body extracts or various isolates, as well as of complex extract mixtures.

## 1. Introduction

Cancer ranks as the leading cause of death overall, while being the first or second leading cause of death before the age of 70 years in 112 of 183 countries [1]. It is known that cancer poses the highest clinical, social, and economic burden in terms of cause-specific disability-adjusted life years (DALYs) among all human diseases, followed by ischemic heart disease and stroke. The overall risk of developing cancer from age 0–74 is 20.2% (22.4% in men and 18.2% in women) [2]. Cancer incidence and mortality is rapidly growing worldwide, which reflects both population aging and growth as well as changes in prevalence and distribution of the main risk factors for cancer. In 2020 alone, 19.3 million new cases and 10 million cancer deaths were estimated. Overall, the five most commonly diagnosed cancers are female breast (11.7%), lung (11.4%), prostate (7.3%), nonmelanoma of skin (6.2%), and colon (6%) cancers. Lung cancer is the leading cause of cancer death (18% of total cancer deaths), followed by colorectal (9.4%), liver (8.3%), stomach (7.7%), and female breast (6.9%) cancers [1].

Cancer is a generic term that designates a large group of diseases that are characterized by sequential and/or simultaneous alteration of molecular pathways associated with cell proliferation, survival, differentiation, and death. Although cancer implies a heterogeneous group of diseases, which differ in the tissue of origin and by the cellular and molecular processes through which they originated, the basic features of tumors were formulated by Hanahan and Weinberg [3], where they defined six basic features common to all tumors, and subsequently expanded them with four more properties that allow tumor progression [4]. The basic six characteristics are the acquisition of the ability for autonomous and unrestricted growth (self-sufficiency in growth signals), avoidance of growth inhibition signals, evading apoptosis, unlimited replicative potential, formation of new blood vessels (sustained angiogenesis), and tissue invasion and metastasis. Additional features include genomic instability and tumor-stimulating inflammation, reprogramming of energy metabolism, and avoidance of the immune system.

Current cancer therapies include sugery, chemotherapy, and radiotherapy, depending on the type and tumor stage [5]. Besides limited effectiveness, there are major problems in treatment, especially with radiotherapy and chemotherapy, which can damage and weaken the patient’s immune system and have numerous other (systemic) side effects such as hepatotoxicity [6], mucositis [7], late gastrointestinal and urogenital side effects, skin damage, exhaustion, and pain that cause a large decline in the quality of life of the patient (QoL), as well as the appearance of secondary tumors [8,9]. Therefore, there is an urgent need for supplementary agents in cancer management and treatment.

While modern scientific research on medicinal mushrooms began during the 1960s in Japan, their traditional medicinal use has been known to exist for about 7000 years in China, India, Japan, and Korea [10]. Mushrooms can be defined as macro-fungi having fruiting bodies that are either hypogeous (underground) or epigeous (above the ground) [11]. Of about 7000 edible mushroom species, around 800 are known to possess pharmacological properties [12]. Medicinal mushrooms are known as a rich source of high- and low-molecular weight bioactive compounds (polysaccharides, polysaccharide-proteins/peptides, peptidoglycans, alkaloids, lectins, lipids, phenolics, polyketides, proteins, steroids, terpenoids, ribosomal, and non-ribosomal peptides etc.), which possess more than 130 therapeutic effects (cytotoxic, mitogenic, immunomodulatory, antiviral, antibacterial, hepatoprotective, hypocholesterolemic, hypoglycemic etc.) [13]. While high molecular weight compounds such as polysaccharides and polysaccharopeptides are primarily known for their immunostimulatory and immunomodulatory action, a large number of species-specific low molecular weight compounds are implicated in direct regulation of cancer signaling, such as nuclear factor-kappa B (NF-κB), mitogen-activated protein kinase pathway (MAPK), Akt, Wnt, Notch, and p53 pathways [11,13]. Due to a large number of pharmacologically active compounds present in certain medicinal mushrooms, they are regarded as potential multi-target therapeutics. This approach is especially important with complex diseases such as cancer, where pleiotropy of cancer pathways is one of the important factors in unsatisfactory effects of certain targeted therapies in the clinic, such as MMP inhibitors, as well as therapeutic resistance [14].

Medicinal mushrooms comprise a complex system of chemical components that have the potential to regulate multiple processes through multiple targets simultaneously. Proteomics is a large scale study of proteins, which is characterized by a hypothesis-free and comprehensive approach to studying novel mechanisms of potential therapeutics. Specifically, differential proteomics, also known as comparative or functional proteomics, studies the changes in proteome in different physiological or pathological states between two or more samples [15]. Cancer proteomics encompasses the identification and quantitative analysis of healthy tissue from neoplasia and can be used to identify markers for cancer diagnosis and treatment (biomarkers), monitoring disease progression, and identifying therapeutic targets. Despite its complexity, proteomics is necessary for accurate characterization of pharmacological action. One gene can potentially produce a large number of protein products, because of differential splicing as well as more than 200 posttranslational modifications that proteins can undergo, which affect their function, stability, and protein–protein and other interactions [16].

The first step of functional proteomics comprises protein extraction from treated cells or animal models, followed by protein separation by two-dimensional gel electrophoresis (2-DE) or two-dimensional difference gel electrophoresis (2DE-DIGE). After comparing and selecting protein spots on the gel, the third step involves their identification by mass spectrometry (MS). Lately, isobaric tags method for relative and absolute quantitation (iTRAQ) has emerged as the most widely used high-throughput technology, which integrates identification and quantification, and makes the analysis of differential proteome easier and more efficient [15]. Another important high-throughput method includes protein microarrays. The last step of differential proteomics is bioinformatic analysis, by which it is possible to map the proteins by their biological and molecular function, cellular localization, protein-protein interactions (PPI), and functional pathways through various available databases.

The aim of this review is to provide a comprehensive overview of the current large-scale proteomic research into antitumor properties of medicinal mushrooms. Besides tumor models, this review also features several important articles on proteomic characterization of the immunomodulating effects of medicinal mushrooms, as well as proteomic analyses of their interaction with various chemotherapy drugs. As in other fields, this area has just recently began to gain momentum. The search was performed through several available databases by using the terms “medicinal mushrooms” and “proteomics” or by combining the term “proteomics” with 38 well-known mushroom genera until August 2021. The articles that consider characterization of mushroom bodies and mycelia by proteomic methods are not included in this review, which covers antitumor effects “in situ”, i.e., from treated cell or animal models. All primary research which fits the given criteria and is included in this review paper is summarized in Table 1.

## 2. Genus *Ganoderma*

*Ganoderma lucidum* (Curtis: Fr.) P. Karst, also called Reishi (Japanese), or Lingzhi (Chinese) is one of the most investigated medicinal mushroom species, which has been used in traditional Chinese medicine for promoting good health, vitality, and longevity for at least 2400 years, when it was recognized by herbalist Shen Nong [17]. This mushroom contains over 400 bioactive compounds, including polysaccharides, nucleotides, sterols, steroids, fatty acids, and proteins/peptides, which have numerous pharmacological effects, such as antitumor, antimicrobial, anti-atherosclerotic, anti-inflammatory, hypolipidemic, anti-diabetic, antioxidative, and radical scavenging, anti-aging, anti-fungal, and anti-viral (for example against herpes and HIV) effects [18].

Over 200 different polysaccharides have been isolated from *G. lucidum* fruit bodies, spores, and mycelia [18]. Mushroom polysaccharides primarily exhibit their antitumor effect through immunomodulation. Polysaccharides from *G. lucidum* can induce cytokine production and differentiation of lymphocytes; maturation of murine bone-marrow derived dendritic cells; and immune response initiated by dendritic cells, proliferation of splenic B cells, and immunoglobulin production and activation of natural killer cells [19]. Polysaccharides isolated from its fruiting bodies contain (1→3) and/or β-(1→6)-d-glucans, α-d-glucans, and polysaccharide-protein complexes, which enhance the cytotoxic activity of natural killer cells and increase TNF-α from macrophages and interferon-γ from lymphocytes. β-d-glucans from medicinal mushrooms induce biological response by binding to membrane complement receptor type 3 (CR3, αMβ2 integrin, or CD11b/CD18) on immune effector cells. The ligand–receptor complex is then internalized, which induces a series of molecular events such as the activation of the nuclear factor NF-κB [20]. A crude extract of the polysaccharides from fruiting bodies induces cytokine expression via Toll-like receptor-4 (TLR-4) modulated protein kinase signaling pathway [21]. Fungal β-glucans act as pathogen-associated molecular patterns (PAMPs) on various immune cell membrane receptors, thus triggering immune function [22]. Large molecular weight polysaccharides have better antitumor efficacy because of their ability to simultaneously bind several receptors. The efficacy of β-glucans also depends on the configuration (triple helix) and the degree of branching.

### 2.1. Ganoderma *spp.* Polysaccharides and Polysaccharopeptides

One of the sources of antitumor polysaccharides are mushroom spores. *G. lucidum* spore polysaccharides induce MAPK pathway and spleen tyrosine kinase Syk-dependent TNF-α and interleukin-6 secretion in murine peritoneal macrophages [21]. Ma et al. [19] demonstrated that *Ganoderma lucidum* spores (GL-SP) could stimulate splenic mononuclear cells (MNCs) proliferation and cytokine production. GL-SP was characterized by high-performance liquid chromatography (HPLC) and seven monosaccharides were identified. MNCs were obtained from inbred KM mice spleen. The proliferation of MNSc treated with 200, 400, or 800 μg/mL of GL-SP for 72 h showed a dose-dependent increase in proliferation. GL-SP also increased the production of IL-2 and TNF-α, although the effect on TNF-α production was more pronounced than on IL-2 production. In order to further investigate the differential protein expression between GL-SP treated (400 μg/mL) and untreated cells, 2-DE was conducted to separate the proteins, and 10 protein spots that exhibited > 2-fold increase or decrease in abundance were further identified by MALDI-TOF MS/MS analysis. Based on their biological functions, these 10 proteins were classified into three categories. Two proteins included in cell viability and proliferation included 14-3-3-tau (theta) protein and apoptosis-associated speck-like protein containing a CARD (ASC), which were both downregulated. Since 14-3-3 tau protein is involved in mitogenesis, cell cycle control (G1-S and G2-M cell cycle progression), and apoptosis, its downregulation may inhibit the apoptosis cascade and increase the number of viable mononuclear cells [23]. ASC protein is essential in intrinsic mitochondrial apoptosis pathway, so its downregulation protects MNCs from apoptosis [24]. Five proteins involved in cell activation and motility were found to be differentially downregulated as a response to GL-SP treatment. Upregulated T-cell-specific GTP-ase plays a role in the activation of lymphocytes induced by GL-SP [25]. Copine I protein, which is involved in apoptosis and TNF-α signaling pathway, was downregulated [26]. Phosphatidylinositol transfer protein α (PITP alpha) modulates cellular responses of lyphocytes to LPS and other mCD14 ligands, so its upregulation may contribute to the immunomodulating activity of GL-SP [27]. Rho, GDP dissociation inhibitor beta, has important roles in the maintenance of marginal zone B cells and retention of mature T cells in thymic medulla, so its upregulation is clearly indicative of its role in immunomodulating effects of GL-SP [28]. Upregulated myosin regulatory light chain 2-A mediates the effect of GL-SP on lymphocyte motility. Three proteins involved in cytoskeleton structure, maintaining cell shape and motility (beta actin, gamma actin, and tubulin alpha), were all downregulated, which could indicate cytoskeletal remodeling in lymphocyte activation [29].

Cyclophosphamide (Cy) is an alkylating agent that is used in treatment of lymphoma, leukemia, ovarian and breast cancers, and small cell lung cancer. Its important side-effect is immunosuppression, which is mediated by excessive free radical production and apoptosis of immune cells of the thymus [30,31]. It has been shown that *Ganoderma lucidum* polysaccharide (GL-SP) also has the potential to at least partly restore immunological effects induced by chemotherapeutic drugs [32]. Ma et al. [33] used 2-DE combined with mass spectrometry to check possible target-related proteins of Cy, as well as thymus protein expression of mice treated with GL-SP or combination of Cy and GL-SP. Proteins whose Cy-induced expression change could be prevented by combined use of GL-SP with Cy were considered as the possible target-related proteins of GL-SP in its mechanism against Cy-induced immunosuppression. Male KM mice were treated either with saline by i.p. injection once daily for 7 days (control group), Cy (20 mg/kg/day, i.p.) for 7 days, GL-SP (50 mg/kg/day, i.g.) for 7 days, or with Cy (20 mg/kg/day, i.p.) and GL-SP (50 mg/kg/day, i.g.) for 7 days. Cy caused significant reduction in body and thymus weight, indicating toxicity and immunosuppression, respectively. GL-SP treatment did not cause a significant difference between body and thymus weight. GL-SP could not fully protect thymus from Cy-induced injury and could partly prevent Cy-induced decrease in proliferation response, but could not fully restore it. In the proteomic study, only the effect of Cy or GL-SP at one dose (20 mg/kg Cy and 50 mg/kg GL-SP) was done, based on the lowest effective dose. Significantly differentially expressed protein spots (*p* < 0.05) with >2-fold difference with respect to control were identified by MALDI-TOF MS/MS. Proteomic study found 15 proteins that were significantly changed in the Cy-treated group compared with control group. These proteins were mainly involved in the regulation of oxidative stress, mitochondrial function, apoptosis, and immune function regulation. The main effect of these changes is immunosuppressive and toxic effects on immune cells mediated by free radical production and apoptosis [31]. The authors classified these proteins into four categories according to the effect of combined use of GL-SP with Cy on Cy-induced expression change; those whose expression change induced by Cy could not be prevented by combined use of GL-SP and Cy: cytochrome b5 outer mitochondrial precursor, hypoxanthine guanine phosphoribosyl transferase 1 (HPRT1), and transaldolase 1; those whose Cy-induced expression level change could only be partly prevented by GL-SP + Cy: phosphatase 2A inhibitor I2PP2A, high mobility group protein B1 (HMGB1), lactate dehydrogenase (LDH), and progesterone receptor membrane component; those whose expression level change induced by Cy could be totally prevented by GL-SP + Cy: PAF acetylhydrolase 1b alpha 1 subunit, glucosidase II subunit beta, GSH-Px, NADH-ubiquinone oxidoreductase 42 kDa subunit, G3PDH and annexin-1. The last category comprises proteins whose Cy-induced expression level change were further enhanced by GL-SP + Cy: nucleolin and elongation factor 2. GSH-Px (glutathione peroxidase) is one of the proteins whose expression level change could be totally prevented by GL-Sp + Cy. It is one of the primary antioxidant enzymes that scavenges hydrogen peroxide and organic hydroperoxides [33]. It has been shown that Cy causes adaptive increase in GSH-Px activity [34]. Platelet-activating factor (PAF) acetylhydrolase is a protein important in immune function. Unregulated PAF signaling can cause pathological inflammation and has been found to be a cause of sepsis, shock, and traumatic injury [35]. Glucosidase II subunit beta is involved in N-glycan metabolism and immune function [33]. NADH-ubiquinone oxidoreductase (complex I) is the first enzyme of the electron transport chain in mitochondria and a main source of reactive oxygen species (ROS) in mitochondria, with important functions in oxidative stress and cell apoptosis [33,36,37]. Glycerol-3-phosphate dehydrogenase I (G3PDH) is a key enzyme in carbohydrate metabolism whose activity is known to be elevated after treatment with Cy with either methotrexate or 5-fluorouracil [38]. Annexin-1 (phospholipase A2 inhibitory protein or lipocortin I) is an endogenous anti-inflammatory protein that modulates innate (neutrophils and macrophages) and adaptive immune response such as TCR signaling and differentiation. It is known to be highly expressed in T cells from rheumatoid arthritis patients [39]. Furthermore, proteins whose Cy-induced expression change could be prevented partially by the combined use of GL-SP with Cy and could also be considered as the possible target-related proteins of GL-SP: phosphatase 2A inhibitor I2PP2A (apoptosis), high mobility group protein B1 (immune function/apoptosis), lactate dehydrogenase (LDH) (cell proliferation/cell death), and progesterone receptor membrane component (apoptosis) [33].

Although previous studies proposed that *Ganoderma lucidum* polysaccharides exert their anticancer effects primarily through immunomodulation, studies have demonstrated other important mechanisms, such as anti-angiogenesis, inhibition of tumor cell motility, induction of apoptosis, and antimutagenic activities [40,41,42,43]. A study performed on a murine sarcoma 180 (S180) model revealed marked protein changes after treatment [44]. *Ganoderma lucidum* polysaccharides (*Gl*PS) were extracted by hot water from the fruiting body. *Gl*PS is a polysaccharide peptide with a molecular weight of 584,900, with a polysaccharide to peptide ratio of 93.51%:6.49%. Male Balb/c mice inoculated with S180 tumor cells were treated with 25, 50, and 100 mg/kg *Gl*PS orally on the second day after inoculation for 10 contiguous days. The tumor growth inhibition was 32.67%, 44.80%, and 45.24% after treatment with the aforementioned concentrations of *Gl*PS, exhibiting a dose response. Proteomic analysis was done from the serum of treated animals. Serum proteins were separated by their isoelectric points and then by molecular mass using sodium dodecyl sulfate polyacrylamide electrophoresis (SDS-PAGE). Three proteins with marked changes in protein profiles were discovered. Serum amyloid A (SAA) was one of the upregulated proteins discovered, the other being haptoglobulin. Apolipoprotein A-II was the only identified downregulated protein. SAA is one of the major acute-phase serum proteins, whose concentration can be elevated 1000-fold in comparison to normal values as a result of inflammation or various malignancies, which suggests its beneficial role in host defense [44,45,46]. SAA inhibits malignant cell attachment to extracellular matrix, (ECM); induces the expression of enzymes, which degrade ECM and stimulates leukocyte recruitment [47,48,49]; and is an important biomarker in several types of malignancies, including gastric, pancreatic, and non-small cell lung cancer [46,50,51]. The hypothesis that one of the main mechanisms of *Gl*PS is the inhibition of tumor cell adhesion was tested by cell adhesion assay. It was shown that the adhesion ability of PC-3M prostate cancer cells to HUVEC endothelial cells was significantly inhibited by *Gl*PS-treated serum. Since it is the same group in which the concentration of SAA was much higher than in control serum, this correlation was interpreted to be of potential functional significance [44].

Sleep disorders are known to be linked to many human body disorders, including cancer. Some clinical studies have shown that sleep fragmentation strongly correlates with tumor metastasis [52]. Xian et al. [53] studied the effects of *Ganoderma lucidum* polysaccharide peptide (GL-pp) on tumor metastasis under conditions of sleep fragmentation. Balb/c nude mice were injected with 5 × 10^6^/mL B16-F10-luc-G5 melanoma luciferase expressing cells through tail vein, which is a common model for studying tumor metastases. Mice were divided into four groups, including untreated control, tumor-bearing group (T group), tumor-bearing group subjected to sleep fragmentation (SF) burden (T + SF group), and T + SF group treated with GL-pp (GL-pp group). GL-pp, with a molecular weight of 512,500 and the polysaccharide to peptide ratio of 94.84%:5.16% was administered i.g. (80 mg/kg) for 15 consecutive days. The survival rate was observed to be equal in all groups (100%). In vivo imaging using luciferase has shown significantly stronger luminescence in T + SF group than in T and GL-pp groups, which indicates an elevated tumor burden. The group treated with GL-pp exhibited a lower luminescence and a decreased number of lung metastatic foci compared with both T and T + SF groups. This indicates that GL-pp has an antitumor metastasis effect under SF conditions. GL-pp also induced M1 macrophage polarization [53]. Label-free quantitative whole proteomics of lung tissues was conducted in order to analyze the differences in protein expression between T + SF and GL-pp groups. Nano-ESI-LC-MS/MS analysis detected 227 genes that were differentially expressed, of which 137 were upregulated and 90 were downregulated. Global gene network analysis based on the KEGG signaling pathway identified 43 key regulatory genes, of which 30 were upregulated and 13 were downregulated. GO biological process analysis of 43 key regulatory genes showed that GL-pp significantly upregulated six biological process clusters: response to hormone, inositol lipid-mediated signaling, glycolipid metabolic process, lipid catabolic process, positive regulation of growth, and morphogenesis of a branching epithelium. Seven upregulated KEGG pathways after GL-pp treatment were: focal adhesion, glycerophospholipid metabolism, choline metabolism in cancer, metabolism of xenobiotics by Cyt P450, purine metabolism, extracellular matrix (ECM)–receptor interaction, and cAMP signaling pathway, while significantly downregulated pathways were herpes simplex infection (KEGG) and mRNA processing (GO). Cytoscape analysis showed that “focal adhesion” clusters, which includes choline metabolism in cancer (mmu05231), PI3K–Akt signaling pathway (mmu04151), and MAPK signaling pathway (mmu04010) as well as “response to hormone” cluster including pathways in cancer (mmu05200), small-cell lung cancer (mmu05222), and chemokine signaling pathway (mmu04062) had the most tight correlations and were situated in the center of the plots. *Lama2* (laminin subunit alpha-2) had the highest degree in the global transduction network, which indicates a strong correlation between *Lama2* and other genes in the signal network. *Lama2* is a tumor suppressor gene, and its decrease in expression is accompanied by an increase in DNA methylation near the transcription site [54]. GO and KEGG analyses revealed that “focal adhesion” cluster had more and tighter correlations among the downregulated pathways, which is in accordance with previous research, which found that the recombinant protein of *G. lucidum* inhibited epithelial to mesenchymal transition (EMT) in LLC1 Lewis lung carcinoma cell line, a process of key importance in tumor metastasis [55]. The other two genes that had tight correlations in the pathway of focal adhesion were Ptk2 (PTK2 protein tyrosine kinase 2 (PTK2), also known as focal adhesion kinase (FAK) and Grb2 (growth factor receptor-bound protein 2). Overexpression of Ptk2 has been shown to improve cell migration, invasion, adhesion, proliferation, and survival in ovarian and other cancers [56]. Grb2 is involved in many oncogenic pathways as an adaptor protein. It was shown that it regulates MAPK and Akt pathways in non-small-cell lung cancer (NSCLC) [57]. Gut microbiota has a strong influence on oncogenesis, tumor progression, and response to therapy [58]. By using 16S rRNA sequencing, Xian et al. [53] also showed that GL-pp treatment decreased Firmicutes:Bacteroidetes (F:B) microbial taxa ratio, which was elevated in the T + SF group. Increased F:B ratio is associated with inflammation and poor prognosis in many diseases [59,60].

Liver cancer, of which hepatocellular carcinoma is the most common, is the third in terms of mortality worldwide [1]. Its complex etiology includes many environmental factors, such as hepatitis B or C viruses (HBV, or HCV), alcohol, and aflatoxin-contaminated food [61]. Recently, a proteomic analysis was conducted to study the effects of various mushroom polysaccharides on hepatocellular carcinoma cells (HepG2) [62]. *Phellinus linteus* (PL), *Ganoderma lucidum* (GL), and *Auricularia auricula* (AA) powders were purified, and single fraction polysaccharides were obtained. HepG2 cells were treated with 1 mg/mL of PL, GL, or AA, and after 2-DE, spots were analyzed by MALDI-TOF-MS for protein identification. It was established previously that these PL, GL, and AA polysaccharides inhibit HepG2 and Bel-7404 cells through the induction of apoptosis (through Bcl-2 activation, increase in mitochondrial cytochrome c, and Smac release) and G1- or S-phase cell cycle arrest (through suppression of Akt, enhancement of p27^Kip^ or p21^Cip^, and suppression of cyclin D1/CDK4 and cyclin E/CDK2) [63]. So, the goal of this subsequent research was to study the effects of mushroom polysaccharides on HepG2 tumor markers [62]. 2-DE analysis revealed a total of 104 differentially expressed protein spots in gels treated with either PL, GL, or AA in comparison to control cells. Differentially expressed proteins *n* = 59 identified by MASCOT analysis were evaluated by MALDI-TOF-MS mass spectrometry analysis. These proteins were subjected to Gene Ontology analysis (GO), by which 400 biological processes (BP) and 146 molecular functions (MF) were found. BP analysis showed that 2.28% of the identified proteins were involved in gene expression process, 1.98% of the proteins were associated with small molecule metabolic processes, and 1.67% with negative regulation of apoptotic processes. KEGG analysis revealed 78 enriched metabolic pathways, which are significant after this treatment, of which the top 10 were: antigen processing and presentation, proteasome, Epstein–Barr virus infection, protein processing in endoplasmic reticulum, glycolysis/gluconeogenesis, RNA degradation, amoebiasis, spliceosome, legionellosis, and pathogenic *Escherichia coli* infection. Authors found that 14-3-3 protein was involved in many KEGG pathways and subsequently confirmed its upregulation with respect to control after treatment with PL, GL, or AA polysaccharides by RT-PCR and Western blot analysis. 14-3-3 was involved in several enriched pathways important in tumor markers and cellular signal transduction, such as Epstein–Barr virus infection pathway, Hippo signaling pathway, viral carcinogenesis pathway, cell cycle pathway, and PI3K-AKT signaling pathway. The upregulation of 14-3-3, which are involved in apoptosis inhibition and tumor genesis and development, was determined to be a possible resistance mechanism of polysaccharide-treated HepG2 cells [62]. The other protein that was key in the networks studied, DJ-1 (protein deglycase DJ-1 or PARK7), was confirmed by RT-PCR and Western blot to be downregulated as a result of treatment in all three groups, which was also in agreement with 2-DE results. DJ-1 has a growth-related function and is upregulated in HCC tissues [64]. Moreover, it is implicated in various mechanisms of inhibiting apoptosis, such as death-inducing signaling complex (DISC) [65]. Therefore, it was concluded that its downregulation might be an important prognostic biomarker for HCC.

### 2.2. Ganoderma lucidum Immunomodulatory Proteins

Mushrooms produce a large number of biologically active proteins including lectins, ribosome inactivating proteins (RIPs), fungal immunomodulatory proteins (FIPs), and laccases [66]. Fungal immunomodulatory protein Ling Zhi-8 (LZ-8) is one of the most important bioactive substances of *G. lucidum* [67]. Lin et al. [68] studied the proteomic profile of LLC1 lung cancer cells after treatment with LZ-8 from *G. lucidum*. It was previously established that LZ-8 has antitumor roles in lung cancer [69], so this study was aimed at revealing the mechanisms of its antitumor action by differential proteomics. C57BL/6 mice were inoculated with 2 × 10^5^ LLC1 cells. Control group was treated with PBS i.p., while the second group was treated with i.p. LZ-8 (7.5 mg/kg) on days 3, 7, 11, and 15. On day 17, tumor tissues were extracted and used for proteomic analysis. After 2-DE separation, protein identification by ESI-MS/MS revealed 21 differentially expressed proteins in comparison with control. Proteins with a value of *p* ≤ 0.05 and fold change ≥ 2 were deemed to be significantly differentially expressed. It was found that significantly downregulated proteins included various heat shock proteins (HSPs), T-complex protein 1, cytoskeleton-related proteins (tubulin, vimentin), protein disulfide-isomerase (PDIA3), and serum albumin [68]. Bioinformatic analysis (Ingenuity Pathway Analysis) revealed that a highly significant overlap of 15 canonical pathways was found and was connected with aldosterone signaling, protein ubiquitination pathways, and 14-3-3-mediated signaling. KEGG annotation revealed that 4 of the 21 proteins, GRP78 (Bip), HSP70, HSP90, and PDI-related proteins were included in protein processing/endoplasmic reticulum stress pathway. Heat shock proteins are a group of chaperone proteins whose expression is often increased in various cancer cells, such as lung cancer [70]. HSP90 stabilizes various oncoproteins, such as EGFR, HER2, ALK, and KRAS, while HSP70 inhibits apoptosis [71]. LZ-8 effectively reduces levels of various HSPs. The tested FIP also inhibited cancer cell viability, suppressed cell migration, and induced apoptosis by HSP downregulation, as was determined by Transwell and Western blot assays. It is known that HSP90 contributes to EGFR stabilization. Previous research demonstrated that LZ-8 effectively downregulates EGFR protein, which supports the finding that HSP downregulation may contribute to cellular apoptotic response [69].

### 2.3. Ganoderma *spp.* Triterpenes

**Figure 1:**
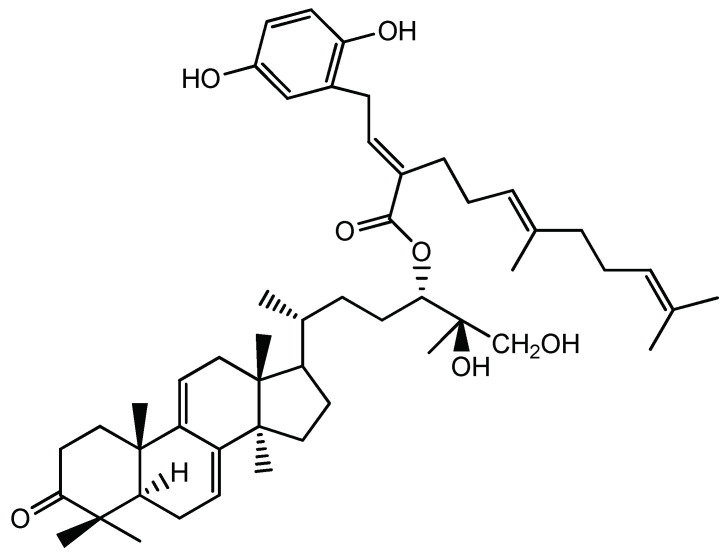
GL22 *Ganoderma lucidum* triterpene.

To date, more than 150 triterpenes from *Ganoderma lucidum* fruiting bodies, spores, and mycelia have been identified. Ganoderic acids (GAs), a group of terpenoids from *G. lucidum*, have anti-inflammatory, anti-tumorigenic, anti-HIV, and hypolipidemic activity [18]. Liu et al. [72] isolated a novel natural triterpene-farnesyl hydroquinone hybrid G22 from fruiting bodies of *Ganoderma leucocontextum* and showed that it significantly inhibits the growth of the liver cancer cell line Huh7.5 in vitro and Huh-7.5-derived tumor xenografts in vivo (Figure 1). Balb/c nude mice were subcutaneously injected with 3 × 10^6^ Huh7.5 cells and daily drug treatment (50 mg/kg/day i.p.) was started when tumor size reached about 100 mm^3^ and lasted for 7 days. Huh7.5 cells were treated with 25 μM GL22 for 0, 12, and 24 h, and protein identification was done by LC-MS/MS, with a fold change cutoff of above 1.3 or below 0.77 deemed significant. G22 and Sorafenib (positive control) strikingly inhibited Huh7.5 xenograft tumor growth in mice, as determined by tumor volumes. Proteomic analysis identified 128 and 141 proteins that were differently expressed (1.3-fold change cutoff and *p* < 0.05) in Huh7.5 cells treated with GL22 for 12 and 24 h, respectively. The authors focused on 12 differentially expressed proteins that are involved in fatty acid metabolism, and which were downregulated after GL22 treatment. GL22 treatment significantly decreased the levels of multiple FABPs (fatty acid-binding proteins). FABPs reversibly bind fatty acids (FA) with high affinity, and FABP content in most cells is generally proportional to the rate of fatty acid metabolism [73]. In concordance with those results, the authors have shown that the levels of peroxisome proliferator-activated receptor components PPARα and PPARγ, which play a crucial role in lipid metabolism, were also significantly decreased after GL22 treatment in vitro. Moreover, it was confirmed that the expression levels of PPARα, PPARγ, FABP1, FABP4, and FABP5 were downregulated in GL22-treated xenograft tumors, which indicates that PPAR-FABPs signaling pathway exerts a significant anticancer effect against liver cancer. Reduced expression of FABPs in vitro and in vivo inhibits FA mobilization and cardiolipin biosynthesis [72]. Metabolic reprogramming is considered a hallmark of cancer [4]. Altered lipid metabolism, especially with regards to fatty acids, is important in cancer cell growth and proliferation, so its reversal demonstrated by GL22 administration points to a significant anticancer effect [74]. GL22 decreased the level of cardiolipin, which is essential for mitochondrial function, which partly explains the antitumor activity of GL22. These effects on cellular lipid homeostasis resulted in altered mitochondrial shape and ultrastructure, which led to mitochondrial dysfunction, including reduced ATP production, decreased aerobic respiration, and increased compensatory anaerobic respiration [72].

Yue et al. [75] studied the effects of ganoderic acid D on the proteome of human cervical carcinoma HeLa cells. GAD was isolated and purified from *G. lucidum* and further purified by HPLC to obtain at least 99% purity. HeLa cells were incubated with 10 μM of GAD for 48 h and separated by 2-DE. Protein spots with 2-fold or more increased or decreased intensity with respect to control were subjected to further identification by MALDI-TOF MS/MS. Cytotoxic effects of GAD on HeLa cells in a range of concentrations from 1–50 μM for 24, 48, and 72 h was observed, proving to be dose- and time-dependent. Since 10 μM was the lowest concentration at which GAD induced both G2/M arrest and apoptosis, this concentration was chosen for protein analysis. Seven downregulated and 14 upregulated protein spots were identified. Proteins including eIF5A (eukaryotic translation initiation factor 5A-1) and spermidine synthase are important in cell survival and proliferation, and their observed downregulation after GAD treatment indicates their possible connection with cell growth inhibition [75]. Contrary to usual findings in both cervical and endometrial carcinoma, the expression of annexin A5 was increased as a result of GAD treatment [76]. Annexins are important in several biological processes, including membrane trafficking, proliferation, differentiation, and apoptosis, and are important positive or negative prognostic biomarkers, depending on the cancer type [77,78]. 26 S proteasome subunit p40.5, which is an important subunit of proteasomes, was increased after GAD treatment, which might contribute to possible protein degradation of HeLa cells. Ephrin receptor EphA7, thioredoxin-dependent peroxide reductase mitochondrial precursor, activator of heat shock 90-kDa protein ATPase homolog 1, ubiquinol-cytochrome c reductase core I protein, protein-disulfide isomerase, aminopeptidase B, and mitofilin are enzymes or regulators of enzymes that play important roles in cell metabolism, and whose change in protein expression indicated changes in metabolism of HeLa cells as a result of treatment. A member of the peroxiredoxin family of antioxidant enzymes PRDX3, which is an important component of antioxidant defense system and mitochondrial homeostasis, was upregulated after GAD treatment of HeLa cells. This indicates its possible role in HeLa cells growth inhibition, since PRDX3 overexpression has been correlated to decreased cell growth [79]. GAD-induced apoptosis may also be induced by several cytoskeleton-related proteins that were found to be downregulated, including microtubule-associated protein RP/EB family member 1, cytokeratin 19, cytokeratin 1, and calumenin. Namely, these proteins participate in cell cycle control and apoptosis, while cytokeratin 19 expression is known to be elevated in cervical carcinoma [75,80]. The authors found that the 14-3-3 family of proteins may have an important role in the cytotoxicity mechanism of GAD, since they were upregulated. Moreover, the identification of potential protein targets for GAD by INVDOCK program revealed that six members of 14-3-3 protein family were predicted to be able to bind directly to GAD.

**Figure 2:**
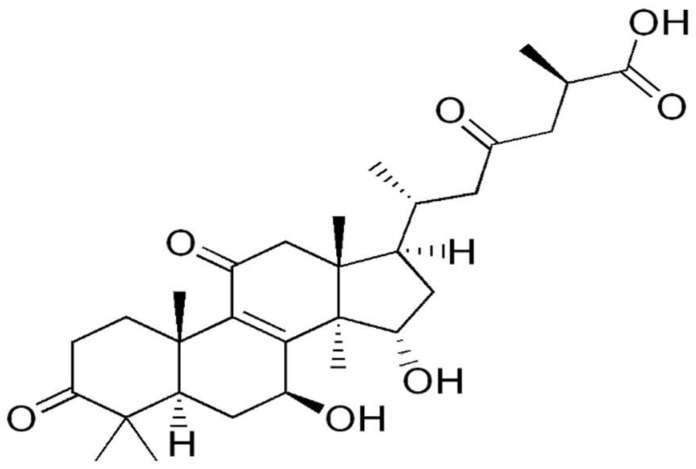
Ganoderic acid A.

Furthermore, Yue et al. [81] subsequently analyzed the proteomic profile of HeLa cells treated with five purified ganoderic acids: ganoderic acid F (GAF), ganoderic acid K (GAK), ganoderic acid B (GAB), ganoderic acid D (GAD), and ganoderic acid AM1 (GAAM1) (Figure 2). The purity of the ganoderic acids was more than 98%. Based on the IC50 value obtained through cytotoxicity assay, which was about 15 μM for all ganoderic acids (GA), HeLa cells were incubated with the aforementioned concentration of either GA for 48 h. Protein spots with 2-fold or more increased intensity and statistically significant in each ganoderic acid-treated group were chosen for identification by MALDI-TOF MS/MS. Among the protein spots that were differentially expressed in each ganoderic acid-treated group, 12 protein spots were found to show similar change tendency in all ganoderic acids-treated groups compared with control. These 12 differentially expressed protein spots were identified by MS/MS. These 12 possible target-related proteins of ganoderic acids could be classified into four categories according to their biological function: cell proliferation or cell death, carcinogenesis, oxidative stress, and calcium signaling and endoplasmic reticulum (ER) stress. Tue same as was discovered in their previous research, one of the downregulated proteins related to cell proliferation and/or cell death was eIF5A, which functions as an elongation factor while 14-3-3 beta/alpha proteins are upregulated [82]. Ubiquilin 2, which modulates proteasome-mediated protein degradation, thus increasing their half-life, was downregulated. PP2A subunit A RP65-alpha isoform, a subunit of PP2A (protein phosphatase 2), which is essential for cell survival, cell cycle regulation, and DNA damage response, was downregulated. Proteins from the second group are the carcinogenesis-related proteins, which are differentially expressed in tumor in comparison with normal cells or tissues. Interleukin-17E, which has a role in T-cell-mediated angiogenesis, and heterogeneous nuclear ribonucleoprotein K (HNRPK), with roles in mRNA splicing and processing, were downregulated as a result of GA treatment [83,84]. Proteins that have important roles in oxidative stress, namely peroxiredoxin 2 (PRDX2) and DJ-1 protein chain A, were both downregulated. Since they both have functions in reducing oxidative stress, it can be hypothesized that they could be involved in ROS-mediated tumor cell death [85,86]. Cancer cells have an increased ROS level compared to normal cells due to high metabolic rate and mitochondrial dysfunction, which render increased susceptibility to oxidative stress [87]. Nucleobindin-1 is a protein involved in ER stress by regulating a function of activating transcription factor 6, an ER membrane-anchored transcription factor [88]. Reticulocalbin 1 is involved in the regulation of calcium-dependent activities in the ER lumen [89]. The authors concluded that eIF5A, 14-3-3 protein, and peroxiredoxin might be the most important target-related proteins of ganoderic acids [81].

Yue et al. [90] also researched possible interactions of triterpenes from *G. lucidum* (GTS) and doxorubicin (DOX). After isolation of GTS from *G. lucidum* fruiting bodies, the main components of GTS were identified using MS. 2-DE separation of proteins was done after treating HeLa cells with 15 μg/mL of GTS for 48 h, which was based on the previously determined IC50 value. The significantly differentially expressed protein spots (*p* < 0.05) with at least two-fold increase or decrease in intensity between control and GTS-treated groups were selected and subjected to further identification by MALDI-TOF MS/MS. *Ganoderma* triterpenes exhibited a weak cytotoxicity against human carcinoma HeLa cells, with GTS being IC50 = 15.4% ± 0.6 μg/mL, while DOX IC50 to HeLa was 31 ± 4.2 μg/mL. By using a combination index method (CI), it was determined that combinations of GTS and DOX had a synergistic effect, since CI values were all below 1. Synergism was also noted between DOX and lucidenic acid (LCN), but LCN was excluded from further studies due to its high IC50 value (86.1 ± 4.2 μg/mL). Proteomic analysis revealed 14 proteins whose expression was significantly altered in the GTS-treated group versus control (untreated) group. In accordance with previous reports [75,81], the eIF5A, PRDX2, PP2A, and cytokeratin 19 proteins were downregulated as a result of treatment with GTS. Triptophanyl-synthetase is one of the important constituents of the early stages of translation, whose expression was also found to be downregulated. 14-3-3 beta/alpha protein, however, was also downregulated, which is not in accordance with [81]. A protein that was found to be differentially expressed, Ran-binding protein 1, can cause mistakes in cell cycle progression [91]. The authors hypothesized that downregulation of 14-3-3 β/α expression and upregulation of Ran-binding protein 1 expression by GTS treatment may cause cell cycle arrest in HeLa cells [90]. Protein proteasome α 1 subunit isoform 1, an important subunit of proteasomes, was downregulated as a result of GTS treatment. It has been shown that proteasome inhibitors have effective antitumor activity in vitro by inducing apoptosis [92]. Along with PRDX2 and cytokeratin, chain B of the Ku heterodimer (Ku80) has important roles in the sensitization of HeLa cells to chemotherapy [90]. Ku80 is a subunit of a DNA-binding subunit of the DNA-PK holoenzyme that has roles in DNA repair and was noted to be downregulated as a result of GTS treatment. It was shown that the decrease in level of Ku can increase the response of cancer cells to DNA-damaging agents [93]. Proteins involved in energy production and utilization, including ATP synthase F_0_ subunit d, enoyl CoA hydratase chain 1, and LDH B, were found to be downregulated as a result of GTS treatment, which may have also indirectly contributed to inhibition of cell proliferation by GTS. This research has also shown that GTS and DOX exhibit synergistic anticancer effects. Besides the combination index which proved synergism in terms of cytotoxicity, flow cytometry analysis demonstrated an increase in the percentage of apoptotic cells in DOX + GTS group, besides G2-M cell cycle arrest. Western blot analysis showed that DOX had no effect on decreased expression of eIF5A or 14-3-3 β/α protein, but showed a slight decreasing effect on the Ku80 level. Measuring intracellular ROS levels showed that DOX has a synergistic effect with GTS in increasing ROS levels, featuring it as an important anticancer mechanism.

## 3. *Lentinus edodes*

This mushroom, widely known as Shiitake mushroom, is known for its powerful antitumor effects via activation of the immune system [94]. The most studied polysaccharide from *Lentinus edodes* is lentinan, a β-(1→3)-d-glucan having two β-(1→6)-d-glucopyranoside branches for every five β-(1→3)-d-glucopyranoside linear linkages. Its molecular weight is 5–15 × 10^5^ Da [95]. An important factor in its immune-stimulating effectiveness is its confirmation, with triple-helical lentinan exhibiting a tumor inhibition ratio of 49.5%, close to a reference anti-cancer drug [66].

### 3.1. Various Lentinus edodes Polysaccharides

Zhang et al. investigated anti-tumor activity of MPSSS, a novel polysaccharide purified from *Lentinus edodes* on cancer-associated fibroblasts (CAFs), which are the essential component of the tumor immunosuppressive microenvironment [96]. A previous study from the same group showed that MPSSS inhibits tumor growth in vivo and can prevent the immunosuppressive function of prostate CAFs [97,98]. Prostate-CAFs were used to prepare conditioned medium with 0, 0.2 mg/mL, 0.4 mg/mL, 0.6 mg/mL, 0.8 mg/mL, and 1 mg/mL of MPSSS for 24 h. CAF medium, which was conditioned without MPSSS, promoted the growth of PC-3 prostate tumor cells, which is consistent with the studies that prostate CAF contributes to tumor development, while the proliferation was inhibited with a rising concentration of the MPSSS-conditioned CAF medium [99]. To better understand the functional molecules on PC-3 cells, the supernatants of prostate CAFs either untreated or treated with MPSSS were separated into high (>100 kDa) (hmwCAFS/MT-hmwCAFS) and low molecular weight secretome fractions (3–100 kDa) (lmwCAFS/MT-lmwCAFS). It was established that while lmwCAFS promoted, MT-lmwCAFS significantly inhibited the proliferation of PC-3 cells. However, no effect was found between PC-3 cells treated with hmwCAFS and MT-hmwCAFS. Therefore, only lmwCAFS and MT-lmwCAFS were subjected by comparative secretome/proteome analysis. lmwCAFS/MT-lmwCAFS and lmwCAFS-treated PC-3 cells/MT-lmwCAFS-treated PC-3 cells were labeled with TMT 6-plex and analyzed by LC-MS/MS. After prediction of genuine-secreted proteins using SignalP, SecretomeP and UniProt 724 of 2909 proteins were predicted as the genuine secreted. For 724 genuine-secreted proteins, 73 proteins were significantly differentially expressed with a *p* value < 0.05 and a fold-change score >1.3 or <0.77. Among them, 44 proteins were upregulated and 29 proteins were downregulated in MT-lmwCAFS compared to lmwCAFS. Heatmap analysis showed that 73 differentially expressed proteins were enriched for chaperone binding and transforming growth factor beta (TGF-β) binding. HscB (iron-sulfur cluster co-chaperone protein HscB) is highly differentially expressed protein (DEP) in chaperone binding, since it acts as a cochaperone of HSP70 and mediates iron-sulfur cluster biogenesis [100]. TGF-β3 is highly DEP in transforming growth factor beta binding. TGF-β3 was strongly upregulated in MT-lmwCAFS compared to lmwCAFS. Highly expressed TGF-β3 has been linked with the inhibition of PC-3 proliferation [101]. DEPs (*n* = 188), including 71 downregulated proteins and 117 upregulated proteins (*p* value < 0.05 and a fold-change score > 1.3 or <0.77), were observed between lmwCAFS-treated PC-3 cells and MT-lmwCAFS-treated PC-3 cells. Heatmap analysis showed that biological processes that were enriched between those groups were cell cycle, regulation of lipid metabolism, response to stress, and response to growth factors. The cell cycle was the most prominent biological process that was altered. KEGG analysis showed that Forkhead box O (FoxO) was the most affected pathway. Since FoxO pathway is regulated by TGF-β, insulin, and AMPK, and the previous results showed that TGF-β3 from MT-lmwCAFS might inhibit cell proliferation, it was hypothesized that TGF-β3 interacts with proteins belonging to FoxO pathway in PC-3 cells. This was confirmed through STRING analysis, where it was found that TGF-β3 interacts with IL-6, SMAD2, and TGFBR2 (TGF beta receptor 2) directly. The direct interaction of p21, Plk1, and cyclin B was also observed. p21 was significantly upregulated and cyclin B and Plk1 were both downregulated in MT-lmwCAFS-treated PC-3 cells compared with lmwCAFS-treated PC-3 cells. Western blot confirmed the upregulation of TGF-β3. The hypothesis that TGF-β3 from MT-lmwCAFS arrests the cell cycle was confirmed by cell cycle analysis, which confirmed that there was a significantly higher percentage of PC-3 cells in G0/G1 phase after treatment with MT-lmwCAFS. Coimmunoprecipitation experiments in PC-3 cells showed that smad3-smad4 complex, which is a mediator of TGF-β pathway, was indeed activated by MT-lmwCAFS and interacted with the FoxO3 protein. In conclusion, TGF-β induces cell cycle arrest via FoxO pathway [96]. This study was in accordance with others that confirm that upregulated TGF-β expression is relevant in tumor cell cycle arrest [102].

### 3.2. Lentinan

**Figure 3:**
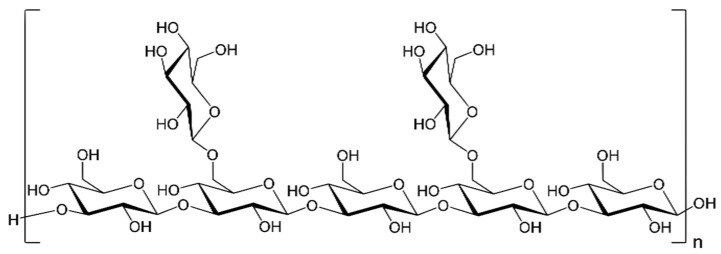
Lentinan structure.

Lentinan is a β-(1→3)-d-glucan, polysaccharide, and a potent anti-cancer drug that has been licensed in Japan for antitumor therapy since 1985 [103,104] (Figure 3). Wang et al. [105] studied the effects of lentinan in the liver cancer model. MTT assay showed that increasing doses of lentinan showed a dose-response decline in the proliferation of H22 cancer cell line, while a cytotoxic effect on normal human liver cell line HL7702 was not observed, indicating a specific effect. The research was continued in a mouse model where KM mice were immunized with either 0.02 mg/kg lentinan i.p. (L1 group), 0.4 mg/kg lentinan i.p. (L2 group), or 1 mg/kg lentinan (L3 group) i.p. once a week for 3 weeks. One week after the third immunization, all of the mice except the control group were injected with 1.5 × 10^6^ H22 hepatocarcinoma cells i.p. Survival analysis revealed that survival rate measured by ILS (increase in lifespan) was 20% in the L2 group while the survival rates in L1 and L3 groups were zero 20 days after establishment of the H22 model. The immunological parameters, namely thymus index and spleen index, revealed a significant improvement i.e., an increase in these parameter values in L2 group in comparison with H22 untreated mice. This indicates a significant improvement in both T-cell and B-cell proliferation rate in L2 group in comparison with control. Macrophage phagocytic indices in L1 and L3 groups were significantly lower than in the control group, while there was no significant difference in phagocytosis rate between experimental lentinan groups and control or model groups. For proteomics, H22 cells were incubated with 1.28 mg/mL lentinan or without it (control group). LC-MS/MS analysis identified six potential protein targets of lentinan. 60S acidic ribosomal protein was increased, which can increase the proliferation rate of cancer cells [106]. Peroxiredoxin 2 has an important role in cancer cell maintenance, through its influences on antioxidative effects on cell survival, proliferation, and apoptosis [107]. Annexin A5 is involved in membrane organization and dynamics, with a possible influence on cell proliferation and invasion [108]. PDZ and LIM domain protein has been shown to promote breast cancer proliferation and metastasis [109]. Both cortactin and moesin proteins are involved in the promotion of tumor invasion and metastasis [110,111]. These results are in accordance with the observation that the highest dose of lentinan in vivo in L1 group promoted the proliferation of cancer cells. The authors concluded that while certain doses of lentinan (L2 group) assist in liver cancer immunoprophylaxis, it must be used with caution.

Lentinan has also been studied in ascites and solid H22 liver cancer models [112] (preprint). H22 cells suspension (1 × 10^7^) was cocultured with 170 μL/mg of lentinan in order to produce an immunogen (liver cancer vaccine LHA). SPF KM mice were treated except for the control group. LHA-1 and LHA-2 groups were treated intraperitoneally (i.p.) with antigens (0.2 mL/mouse) once per week for 3 weeks. One week after the third immunization, all of the mice were inoculated with 1.5 × 10^6^ of H22 cells. Model-1 group and LHA-1 group were inoculated with H22 cells (1.5 × 10^6^) i.p. to establish the H22 ascitic tumor model, while model-2 group and LHA-2 group were inoculated with H22 cells *s.c.* to establish a solid tumor. Unlike model-1 and model-2 mice, both LHA-1 and LHA-2 treated groups had a similar body weight to control group, which was significantly lower than in model-1 and -2 mice, which indicates an antitumor effect. Antigen protein sample was analyzed by LC/MS, and 32 dysregulated proteins were found. Out of the 32 new proteins, 6 were found to have an antitumor effect by UniProt. Their main functions are apoptosis mediated by TNF-α-related apoptosis-inducing ligand, inhibition of angiogenesis, and direct inhibition of invasion and metastasis. Calreticulin (CRT) is a molecular chaperone that regulates Ca^2+^ homeostasis, cell adhesion, and gene expression. Septin-7 (SEPT7) inhibits matrix metalloproteinases and upregulates their negative regulators TIMP, and can therefore inhibit tumor growth and metastasis [113]. Brain-specific angiogenesis inhibitor 1 can inhibit angiogenesis, which is imperative for cancer growth, progression, and metastasis. YAP1, a transcriptional coactivator, has a tumor suppressor role in many tumors, such as head and neck tumors and tissues [114]. Src substrate cortactin, when overexpressed, can lead to activation of invadopodia, which can ease the spread of cancer to other tissues [115]. Furthermore, this research demonstrated that the LHA vaccine enhances T lymphocyte cytotoxicity and promotes maturation of dendritic cells (DCs) [112].

A similar study on lentinan as an immunogenic cell death (ICD) inducer was done by Wang et al. [116]. It is known that some chemotherapy drugs and radiotherapy can enhance the transformation of tumor cells in apoptosis stage from non-immunogenic to immunogenic cells and activate the immune system by inhibiting various immunosuppressive tumor networks, thus strengthening the antitumor response in the body. This phenomenon is called immunogenic cell death (ICD) [117]. Tumor cells release specific proteins that are characteristic of ICD and feature as danger signals, or DAMPs (danger-associated molecular patterns) including calreticulin (CRT), extracellular adenosine triphosphate (ATP), heat shock protein (HSP), and high mobility group protein B1 (HMGB1) [118]. DAMP are expressed on damaged or stressed cells and are recognized by antigen presenting cells (APCs) in the body, which then activate the adaptive immune response [119]. 1 × 10^6^ H22 liver cancer cells/mL were injected, and about a week later when volume increase was visible, ascites was collected to prepare H22 suspension for ELISA. MTT assay confirmed a dose-dependent decrease in cell viability, while flow cytometry analysis revealed a significant and time- and dose-dependent increase in late apoptotic cells following incubation with various concentrations of lentinan. HPLC-MS analysis revealed that lentinan induces an increase in ICD marker proteins, which confirmed previous ELISA results. Namely, HMGB1, CRT, various heat shock proteins (HSP 90-alpha, HSP 90-beta, HSP 70), ATP (ADP/ATP translocase 2), annexin 1, and ER membrane protein complex subunit 1 were discovered to be differentially expressed (upregulated) as a result of treatment. In conclusion, an increase in immunogenicity and reduction of the immune tolerance mediated by lentinan have been established, which may prove significant in future development of liver cancer vaccines.

Liu et al. [120] studied the effects of lentinan (LNT)-functionalized selene nanoparticles on malignant ascites. Ovarian malignancy is the most common cause of malignant ascites, which is why OVCAR-3 human ovarian cancer cells were used in this study, along with EAT, a common tumor ascites model [121]. Chemotherapy drugs can cause pyroptosis, an inflammatory cell death type, accompanied by various cytokines that may stimulate ascites production [122,123]. Thus, the induction of apoptosis as an immunologically silent process could be helpful for clearing tumor cells and ascites [124]. Selenium nanoparticles (SeNP) are characterized by their biocompatibility and efficacy in comparison to inorganic or organic Se compounds. Selenium itself has anticancer effects [125]. LNT-functionalized SeNP (referred as Selene afterwards) was obtained using Na_2_SeO_3_ and LNT under reduction conditions. EAC malignant ascites model was used in Balb/c mice to study the effects on ascites suppression. The mice were administered Selene 4 days after the EAC model was established. It was shown that Selene could inhibit the ascites and decrease body weight, volume of ascites, and EAC cell numbers, while the use of lentinan (LNT) or SeNP alone affected these parameters only slightly. In a model where Selene was administered only after 7 days after EAC inoculation, ascites could not be suppressed. Another in vivo model used was orthotopic OVCAR-3 model, which was established by implanting cells into athymic nude mice. In this model, Selene could not only reduce ascites, but also induce apoptosis in cancer cells in the ascites. Moreover, Selene-treated mice were only mildly hemorrhagic (vascular leakage), which further confirmed that Selene could inhibit ascites production in this model. Importantly, it was shown that Selene reduced the expression of various inflammatory cytokines, such as IL-1β, IL-6, and TNF-α. Proteomic analysis was done in OVCAR-3 cells after treatment with Selene, combining the SDS-PAGE and LC-MS/MS analysis. Proteins that were significantly differentially regulated (I logFC I > 1.5) were further analyzed. GO and KEGG enrichment analysis revealed that the main functions and signaling pathways affected by Selene included oxidative phosphorilation, endocytosis, apoptosis, adhesion, mitochondrion, and mitochondrion translation. Pharmacological research confirmed that Selene specifically internalizes into mitochondria, where it leads to apoptosis through TLR4/TRAF3/MFN1 pathway. It was shown that LNT can disperse SeNPs and enhance the targeting of SeNPs to tumor cells. In contrast to SeNP, which induced pyroptosis because of being taken up by lysosomes, it was shown that Selene was mainly taken up by the mitochondria, thereby inhibiting inflammatory cytokine secretion in ascites. The authors concluded that Selene is effective for treating malignant ascites and may be developed as a clinical drug [120].

## 4. Genus *Cordyceps*

**Figure 4:**
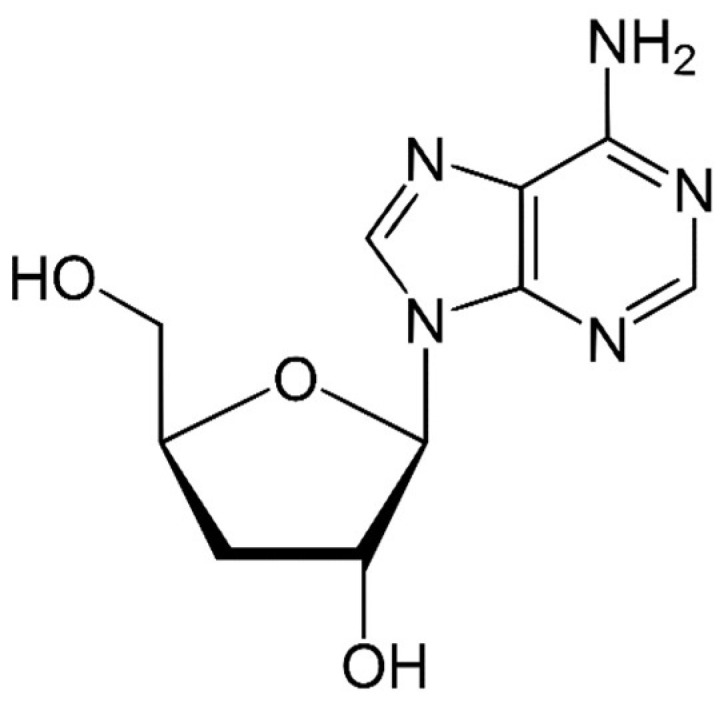
Cordycepin structure.

Species of the genus *Cordyceps* are entomopathogenic Ascomycete mushrooms, which are widely used in traditional medicine. Various Cordyceps species have been identified for their pharmacological properties, including *C. sinensis*, *C. militaris*, and *C. pruinosa*. Some of the studied active ingredients include cordycepin, cordycepic acid, sterols (ergosterol), nucleosides, and polysaccharides [126]. Cordycepin, or 3′-deoxyadenosine, has significant antitumor activities, which include inhibition of cell proliferation, migration, and induction of apoptosis [127,128] (Figure 4). Its effects are dose-dependent; at low doses, it interferes with mRNA production and assembly of proteins, thus inhibiting uncontrolled cell growth and division. At higher doses, it inhibits cell adhesion and blocks protein synthesis through its effects on Akt and 4EBP phosphorylation [129].

Jeong et al. [126] studied the effects of *Cordyceps militaris* fresh fruit bodies or mycelia on cisplatin-resistant A549/CR lung cancer cells. Cisplatin is usually the first-line chemotherapeutic for patients with advanced NSCLC (non-small cell lung carcinoma). Besides its high toxicity, one of the major clinical problems is cisplatin resistance. In this research, cordycepin was first quantified in the sample by HPLC. Qualitative components were further detected by LC-MS. Cell-viability assay showed a dose-dependent inhibition of A549/CR cell viability, with a IC50 = 0.57 ± 0.12 mg/mL. Afatinib, a tyrosine kinase inhibitor, which was used as a positive control, had IC50 = 2.3 ± 0.23 μg/mL. Flow cytometry revealed that time-dependent percentages of the live cells were decreased from 78.32 to 17.63, which was shown to be the consequence of elevated initiator caspases-8 and -9, and executioner caspase-3, and was more prominent in cordycepin (CME) vs. afatinib-treated cells. Cell-cycle analysis demonstrated that CME increased the percentage of sub-G1 A549/CR-treated cells to 33.2 ± 1.2%, compared with Afatinib (17.6 ± 0.6%) and vehicle-treated control group (13.3 ± 1.6%) at 48 h. This demonstrated that CME increased accumulation in S phase in A549/CR cells, which was followed by cancer cell apoptosis. Proteomic profiling was done using a protein chip-based antibody array after treatment of A549/CR cells with 1.5 mg/mL CME. Proteins having a normal median ratio in the range of 1.0 were considered as unchanged expression. Among the cell-cycle proteins analyzed (42 of them), the only protein that exhibited a change in expression levels was H-Ras, which was significantly downregulated. It is well-known that H-Ras is a crucial protein that promotes cell proliferation by regulating cell cycle progression in most cancers [130]. In conjunction with previous results, it is clear that CME controls cell cycle progression by inhibiting Ras downstream signaling, such as Raf/MEK/ERK and PI3K-Akt, which results in suppressing the proliferation of A549/CR cells. The authors suggested that CME showed the possibility of overcoming the cisplatin resistance in NSCLC [126].

Cai et al. [131] investigated the effects of *Cordyceps sinensis* extracts on 4T1 breast cancer cells in vitro and in vivo. It was determined that one of the major components of water extract of *Cordyceps sinensis* (WECS) is polysaccharide, which constitutes 19.83% of extract (*w*/*w*). Nucleosides were also among the main components of WECS, and their content was analyzed by HPLC. Cytotoxicity assay showed that WECS had a significant effect on reducing 4T1 cell viability, being approximately 50% at 0.40 mg/mL. Since metastases present one of the main problems in clinical cancer management, the anti-metastatic effect of WECS was studied specifically. In the metastasis model, 1 × 10^6^ 4T1 cells were injected into the tail vein of Balb/c mice. From the day of tumor inoculation, the mice were intraperitoneally injected with 50 mg/kg/day WECS or vehicle for 15 days. Kaplan–Meier analysis, which was followed up for 33 days post 4T1 injection, showed a significant improvement in mice survival treated with 50 mg/kg/day of WECS. The number of metastatic lung nodules was also significantly reduced in the same group (about 10 per mice), both in their size and number, compared with untreated control (about 70 nodules per mouse). Further evidence of anti-metastatic effect is the 50% reduction in MMP-9 concentration after administration of WECS. Protein analysis of the lung tissue homogenates was done by protein array, which compared the expression of 111 cytokines in treated and untreated 4T1 tumor-bearing mice. It was shown that 6 cytokines were upregulated more than 2-fold in 4T1 tumor-bearing mice compared to normal mice, namely OPN (osteopontin), CCL12 (chemokine (C-C motif) ligand 12), IL-33, CCL17, CCL6, and MMP-9. Of these OPN, IL-33, CCL17, and MMP-9 were significantly reduced in the lung of 4T1 tumor-bearing mice treated with WECS. It is known that various cytokines as well as growth and inflammatory factors have an important role in migration and colonization of metastatic tumor cells [132]. It is known that OPN can promote lung metastasis [133]. IL-33 is also known to promote breast cancer metastasis through increasing immunosuppressive cells [134]. CCL17 is important for homing of CCR4 positive regulatory T cells in the lungs [135]. The authors speculated that WECS can reduce DNA damage and DNA damage response (DDR), which is strongly correlated with immune response and promotes inflammation in late tumor stages through cytokine recruitment, which is in line with previous research on *Cordyceps sinensis* [136].

Wang et al. [137] analyzed the effects of *Cordyceps sinensis* treatment on the liver proteome of rats in which the hepatocellular carcinoma was induced by diethylnitrosamine (DEN). *Cordyceps sinensis* powder was used to prepare an extract that was analyzed with HPLC. The qualitative analysis revealed three major compounds: uridine, adenosine, and ergosterol. Male Lewis rats were divided into two groups. In the DEN-treated group, rats were fed an oral aqueous solution 100 parts per million (ppm) DEN for 120 days through their drinking water. In the DEN/*C. sinensis* group, animals were treated with DEN as before and with an ethanol extract of *C. sinensis* (12.5 mg/kg) via oral gavage for 120 days. It is known that DEN treatment disturbs normal redox balance and shifts hepatocytes into a state of oxidative stress, which includes the carbonylation of proteins that are involved in the progression of liver tumors [138]. The hypothesis of the study was that *C. sinensis* could attenuate the oxidative stress induced by DEN and protect the liver from hepatoma. The hepatoprotective effects were first established by the effect of *Cordyceps* on the levels of liver enzymes, which can serve as markers of hepatic injury. The results have shown that *C. sinensis* almost completely abolished the increase of serum ALT (alanine transaminase) and AST (aspartate transaminase) at 17 weeks, which suggests that *C. sinensis* can effectively inhibit DEN-induced liver cell damage. *C. sinensis* application could also restore histopathological liver changes such as fibrosis at 2 and 8 weeks, which was evident in the vehicle group, as well as the disappearance of B23 (nucleophosmin), which is obvious in altered hepatic foci in the vehicle group. High-resolution 2-DE, together with MALDI-MS, was used to reveal the global protein changes. Thirty proteins with significant changes were identified and, using Map Editor, a network of interactions was made. It was established that DEN disturbed the redox balance and sequentially activated ubiquitin-proteasomal proteolysis cascades responsible for cellular stress responses and promoted the expression of Akt, which elicits strong effects on cell proliferation. The same analysis applied to *C. sinensis*-treated group revealed that it attenuated DEN-induced ubiquitin and inhibited c-Myc, AKT, p53, and NF-κ levels, while activating PPARγ. Functional network analysis by MetaCore revealed that the most significantly enriched processes after treatment with *C. sinensis* were response to hypoxia and oxidative stress, manganese transport, and unfolded protein response. This included a significant changes in expression of several ROS-related proteins, including catalase, DHE3 (glutamate dehydrogenase 1), PRDX1 (peroxiredoxin-1), GSTP (glutathione S-transferase P), GSTM1, and GSTM2 (glutathione S-transferase Mu 1 and 2). From these results, it is clear that *C. sinensis* eliminates ROS, resulting in a significant reduction of the level of redox state-correlated proteins [137]. 2-DE oxyblot analysis demonstrated that *C. sinensis* could reverse protein carbonylation induced by DEN. This is extremely important since DEN carboxylated chaperone proteins and enzymes such as HSP7C (heat shock cognate 71 kDa protein), GRP75 (75 kDa glucose regulated protein), GRP78 (78 kDa glucose regulated protein), propionyl-CoA carboxylase, catalase, and alpha enolase result in the promotion of hepatocellular carcinoma through oxidative stress and protein misfolding. Further chemopreventive effects were demonstrated by upregulation of Nrf-2 (nuclear factor erythroid-derived 2-like 2), which regulates the expression of antioxidant proteins that protect against oxidative damage such as HO-1 (heme-oxygenase-1). Simultaneously, it was evident that *C. sinensis* abolishes c-Myc overproduction caused by DEN, along with inhibiting AKT/mTOR cascade. The PPARγ upregulation regulated lipid metabolism and cell inflammation, which also has an antitumor effect [139]. In conclusion, *C. sinensis* could exhibit HCC-preventive efficacy through various antioxidant pathways, which consequently maintain the stability of various proteins and suppress the oncogenes [137].

Wang et al. [140] investigated anticancer mechanisms of *Cordyceps cicadae* against hepatocellular carcinoma in vitro using a proteomic approach. A lyophilized hot water extract of wild-type *C. cicadae* was used to treat MGCC97H cells in various concentrations (0–1000 μg/mL), and dose-dependent inhibition was observed. Cell cycle analysis revealed that treatment with *C. cicadae* (at 100, 250 and 500 μg/mL) induced a G2/M cell accumulation and decreased the cell percentages in G0/G1 phase. The highest dose (1000 μg/mL) induced a G2/M arrest in 64.75% of the cells. After incubation of MHCC97H cells with 500 μg/mL for 48 h, cells were subjected to proteomic analysis. 2-DE revealed 28 proteins with significant (*p* < 0.05) changes of >1.5 fold in volume intensity, which were selected and identified by MALDI-TOF-MS/MS. The major biological functions of these proteins are cell growth and cell cycle regulation, anti-cancer effects, and other functions (cell redox regulation, protein folding, mRNA splicing). 14-3-3 gamma is one of the proteins that regulates the cell cycle, and its downregulation is contrary to its usual overexpression in HCC [141]. 14-3-3 downregulation could also account for G2/M phase arrest. BUB3 (mitotic checkpoint protein BUB3 isoform A), DCTN2 (Dynactin subunit 2), and MAPRE1 (microtubule-associated protein RP/EB family member 1) are involved in spindle checkpoint and mitosis regulation, and their deregulation may also contribute to G2/M phase arrest [140]. GLRX (thioredoxin-like protein) and CLIC1 (chloride intracellular channel protein 1) also regulate cell growth so their dysregulation could account for G2/M phase arrest. Among the proteins with anti-cancer effects, the upregulation of HSPB1 (heat shock protein beta-1) could indicate some resistance of the cancer cells and the absence of apoptosis in the cells treated with *C.cicadae*. Upregulation of ENO1 (alpha-enolase isoform 1) can result in the promotion of hepatocellular carcinoma through oxidative stress and protein misfolding [142]. ERP29 (Protein-Disulfide Isomerase Related Chaperone Erp29) is active in the endoplasmic reticulum, so its reduced expression can be attributed to reduced ER stress after treatment with *C. cicadae.* ER stress and unfolded protein response are involved in HCC development, aggressiveness, and response to treatment [143]. Downregulation of STRAP (WD-40 repeat protein), which is involved in pre-mRNA splicing, has a positive prognostic significance, because its overexpression was reported in several cancers [144]. Another protein that was downregulated as a result of treatment with *C. cicadae* is peroxiredoxin 1 (PRDX1). The elevated expression of PRDX1 was found in various cancers and its downregulation might facilitate a failure of the endogenous antioxidant systems, which protect cancer cells from ROS [145].

## 5. Genus *Pleurotus*

Mushrooms of genus *Pleurotus*, or oyster mushrooms, comprise about 40 species of lignocellulosic mushrooms. Recently, various molecules with pharmacological properties have been identified, including those with anti-neoplastic and immunomodulating effects (including α- and β-glucans, lentanin, resveratrol, POMP-2 (*Pleurotus ostreatus* mycelium polysaccharide 2), POPS-1 (polysaccharide obtained from the fruiting body of *Pleurotus ostreatus*), concavalin A, cibaron blue affinity protein), and antioxidant activity (pleuran, ergosta-7). The most studied species include *P.ostreatus*, *P. eryngii*, *P. nebrodensis*, *P. citronopileatus*, and *P. sajor-caju* [146].

Finimundy et al. investigated the antitumor, specifically cell-death-inducing properties of *P. sajor-caju* on colorectal cancer model [147]. *Pleurotus sajor-caju* fruiting bodies extracts were obtained using various solvents [n-hexane, chloroform, ethyl acetate, ethanol and ethanol/water (1:1, *v*/*v*)]. HCT-116 colon adenocarcinoma cell line can be classified as a consensus molecular subtype 4 (CSM4, mesenchymal). According to this classification, which is of importance in preclinical research, CSM4 tumors are those that are diagnosed at more advanced stages (III and IV) [148]. In this research, HCT-116*^wt^*, *^-Bax^*, *^-p21^*, and *^-p53^* were used in order to correlate the observed anti-proliferation activity to activation of pro-apoptotic and/or cell arrest regulation pathways. Also, MRC-5 healthy lung fibroblast cell line was used in order to verify the cell selectivity of the treatment. n-hexane extract of *Pleurotus sajor-caju* (PSC-hex) was chemically characterized by GC-MS. The viability assay (MTT) confirmed that the most significant results were obtained with n-hexane extract (PSC-hex) on HCT-116*^wt^* cells (IC50 = 0.05 mg/mL), followed by the PSC acetone extract on the same wild type cell line. Meanwhile, n-hexane extract showed practically no anti-proliferative activity on the MRC-5 cell line. The authors hypothesized that this selectivity might be explained by inhibition of the mitochondrial complex I, which is a target of lipophilic compounds that are extracted by n-hexane. On the other hand, no anti-proliferative activity was observed in Bax (HCT-116*^-Bax^*), p21 (HCT-116*^-p21^*) or p53 (HCT-116*^-p53^*) deficient cell lines, indicating that PSC-hex promotes its cytotoxicity by inhibiting tumor-associated signaling pathways. Flow cytometry analysis showed that, after treatment with 0.05 mg/mL of PSC-hex, the number of HCT-116*^wt^* cells in early apoptosis increased from 0.45% to 65.6%, while the number of viable cells decreased from 92.2% to 25.9%. Furthermore, cell cycle analysis has shown that PSC-hex induces G2/M cell cycle arrest, and a significant accumulation of cells in the sub-G1 fraction, which indicates apoptosis induction. Therefore, it was assumed that PSC-hex exerts the observed cytotoxicity through a pro-apoptotic pathway. PSC-hex caused a loss of mitochondrial membrane potential (ΔΨm), which is followed by cytochrome c release and the activation of caspase-9, which indicated an internal apoptosis pathway activation. Many chemotherapeutics are selectively toxic to tumor cells because they increase oxidative stress i.e., ROS levels in tumor cells (which are already characterized by higher ROS levels than normal cells) above the levels that antioxidant cell mechanisms can resolve [149]. Flow cytometry using DCF-DA stain showed that treatment with PSC-hex causes 3-fold (0.025 mg/mL) and 2-fold (0.05 mg/mL) increase in H_2_O_2_ and O_2_^•−^ levels, respectively. Proteomic analysis by Proteome Profiler Array (43 proteins studied) revealed that there was an increased expression of several apoptosis-related proteins, including Fas, HSP60, HSP70, Xiap, HTRA, Survivin, Smac, caspase-3, cytochrome-c, p52, Bax, Bad, Bid, and Bim. Docking simulation demonstrated that one of the main identified compounds in the PSC-hex extract, ergosta-5,7,22-trien-3β, fits into the Bcl-2 hydrophobic cleft, indicating a possibility of an alternative pathway for inducing apoptosis by direct compound interaction.

Ma et al. [150] analyzed the proteome alterations in RAW 264.7 macrophages as a result of treatment with PEP 1b, a novel immunoregulatory protein isolated from *Pleurotus eryngii*. A previous study obtained and identified this protein (PEP) with a molecular weight of 21.9 kDa and informed that it could boost cellular immune response through cytokine and NO (nitric oxide) secretion. This study identified Toll-like receptor 4 (TLR4) as the receptor for this protein [151]. RAW 264.7, a murine macrophage cell line, was pretreated with medium and then treated with various concentrations of PEP 1b (0, 50, 100, and 200 μg/mL). The overall assessment of proteomic regulation in the PEP 1 b treatment group vs. control group was done using iTRAQ-based protein quantification approach. Three comparison groups were established: PEP 50/CT, PEP 100/CT, and PEP 200/CT, which represented the differences in expression of reliable proteins between 50, 100, and 200 μg/mL PEP 1b treatment groups and control group, respectively. A total of 2277 reliable proteins from RAW 264.7 macrophage cells were identified. Differential proteins were those with an average fold change (FC) of more than 1.2 or less than 0.83 in treated groups compared to control. The numbers of detected differential proteins (DEP) increased with PEP concentration; PEP 50/CT group: 116 proteins (57 upregulated and 59 downregulated), PEP 100/CT group: 165 proteins (89 upregulated and 76 downregulated); and PEP 200/CT group 292 proteins (191 upregulated and 101 downregulated). The expression level of some proteins was stepwise increased with the concentration increase of the PEP 1b treatment, which points to a dose-response relationship: macrophage migration inhibitory factor (Mif), interferon-induced transmembrane protein 3 (Ifitm3), cyclooxygenase 2 (Cox2), Ras-related protein 1b (Rap1b), and sequestosome 1 (Sqstm1). The further analysis of the protein data was focused on PEP 200/CT group. On the basis of GO biological process analysis, differential proteins were mainly attributed to small-molecule metabolic process (18%), immune system process (15%), cellular catabolic process (14%), oxidation reduction process (13%), and inflammatory response (5%), which were associated with the immune-boosting activity of the macrophage. KEGG analysis via heat maps demonstrated that there were four pathways that were upregulated with the increase of the concentration of PEP 1b treatment: immune system, transport and catabolism, carbohydrate metabolism, and signal transduction. KEGG analysis showed that PEP 1b could upregulate immunoregulatory pathways, such as NF-κB, VEGF, and TNF pathways, but also the following pathways: hedgehog signaling pathway, sphingolipid signaling pathway, Rap1 signaling pathway, Wnt signaling pathway, phospholipase D signaling pathway, PI3K−Akt signaling pathway, and Ras signaling pathway. The proteins associated with the NF-κB signaling that was upregulated was Sqstm1, which functions as an adaptor protein in concert with TNF receptor-associated factor 6 to regulate the activation of NF-κB in response to upstream signals [152]. Upregulated Cox2 can also positively regulate the NF-κB nuclear transfer process to mediate the immune response of macrophages [153]. PEP 1b also upregulated the expression of Mif (macrophage migration inhibitory factor), Rap1b (Ras-related protein Rap-1b), transmembrane glycoprotein NMB (Gpnmb), superoxide dismutase (Cu−Zn) (Sod1), C5a anaphylatoxin chemotactic receptor 1 (C5ar1), and peroxiredoxin 2 (Prdx2), which modulate the MAPK pathway. Mif is important in cell-mediated immunoregulation and inflammation as well macrophage function through suppression of anti-inflammatory effects of glucocorticoids [154]. Rap is a protein of the Ras family that affects T cells through integrin and modulates cell adhesion [155]. Peroxiredoxin 2 is a member of antioxidant enzymes that reduces hydrogen peroxide and alkyl hydroperoxides, and activates MAPK signal pathway [156]. The proteomic analysis also revealed that PEP 1b modulated the nitric oxide biosynthetic process through upregulation of Cox2, heat shock protein (Hsp90aa1), Pyk, and Itgb2 (integrin-beta 2). Pyk2 (protein tyrosine kinase 2 beta) is involved in multiple immune signaling pathways (JNK/MAPK, Akt/MAPK, and JNK/SAPK) [157]. This research thus demonstrated that PSP 1b protein can influence critical proteins important in immunoregulatory activities in macrophages.

## 6. *Trametes versicolor*

Turkey-tail mushroom or cloud mushroom, *Trametes versicolor* (or *Coriolus versicolor*), is a mushroom used as an immunomodulatory agent and as a cancer adjuvant, with successful clinical outcomes [158]. *T. versicolor* contains two proteoglycan fractions with anticancer properties: krestin polysaccharide (PSK) and polysaccharidopeptide (PSP). It is known that polysaccharides can reach a greater level of complexity when bound to polypeptides and proteins [66]. Various studies confirmed that they can inhibit adhesion and invasion in cancer cells, cause cancer cell motility, suppress angiogenic factors, as well as induce apoptosis in various types of cancer [159,160,161,162].

Lee et al. [163] analyzed the proteome of human T lymphocytes after treatment with cyclosporine A (CsA) and polysaccharopeptide (PSP) in the context of analyzing their specific properties in restricting the proliferation of activated human T cells. This is of importance in regulating T lymphocyte proliferation in organ transplant recipients and in autoimmune diseases. Cyclosporine A (CsA) isolated from fungus *Tolypocaldium inflatum* is used in the prevention of graft-versus-host disease and the treatment of allograft rejection and certain autoimmune disorders [164]. CsA acts by blockage of IL-2 expression, which inhibits T cell clonal expression and differentiation. However, long term CsA administration can induce cancers, nephrotoxicity, hypertension, and hyperlipidemia [165]. Polysaccharopeptide (PSP) derived from *Coriolus versicolor* was found to possess similar immunosuppressive effects by suppressing IL-2, which reduces the production of IL-2 receptor, cytotoxic T cells, natural killer cells, and gamma-interferon [163]. However PSP has also been reported as a potent immunomodulatory agent against neoplasms and infections [166,167,168]. Ex vivo (healthy male donors, age 20–40) T cells were primed with 5 μg/mL phytohemagglutinin (PHA). CsA (1000 ng/mL) or PSP (500 μg/mL) were given at the same time as PHA. Control cells were set up in the same medium. Proliferation assay using BrdU demonstrated significant suppression of the stimulation index in T cells after treatment with either CsA or PSP. Neither CsA or PSP were able to induce apoptosis in T cells. Both CsA and PSP significantly reduced the production of IL-2. 2DE-electrophoresis revealed approximately 500–550 gel spots on control, PHA, PHA + PSP, and PHA + CsA-treated T cells. There was no significant variation between the resting and PHA-primed cells in the presence of CsA or PSP. MALDI-TOF MS analysis identified 17 proteins with significant expression after treatment with CsA and PSP as compared with PHA treatment alone. Many T cell proteins whose expression was elevated by PHA returned to their baseline level or were further reduced after CsA or PSP administration. Four proteins were significantly affected by both CsA and PSP. Rho GDP dissociation inhibitor β, which functions as a metabolism inhibitor that prevents GDP/GTP exchange reaction, was upregulated, as well as ENO1 (alpha-enolase), which is involved in glucose metabolism and immune cell migration [169,170]. Proteasome and triosephosphate isomerase proteins in the PHA-stimulated T cells were downregulated after CsA or PSP treatment. It is known that proteasome mediated degradation pathway is required for IL-2 signaling [171]. TIM (triosephosphate isomerase) has a role in glycolytic and other metabolic pathways required for efficient energy production. The only protein that was significantly affected by CsA was galectin-1. Galectin-1 upregulation is in line with CsA immunosuppressive functions, since this protein is an antiinflammatory agent that triggers the homeostatic signals, which inhibit lymphocyte effector functions [172]. Three proteins were significantly affected by PSP only. The upregulated ferritin reduces the availability of free iron required for aerobic metabolism, which can mediate PSP-induced effects on T cell proliferation [173]. The downregulated 70 kDa heat shock protein (HSP70) is a molecular chaperone that ensures correct protein folding, so it was hypothesized that PSP might exert some mitotic/meiotic disturbance via HSP70 reduction. The downregulation of peroxiredoxin, which is an antioxidant protein, can cause intracellular accumulation of H_2_O_2_ that eventually affects cell cycle progression [174]. In conclusion, the proteins that were identified in this research may be of further use for monitoring the efficacy of CsA immunotherapy, as well as potentially include PSP to be used as well.

## 7. *Hericium erinaceus*

This edible mushroom, also known as lion’s mane mushroom, is a source of compounds with antioxidant, hypolipidemic, hemagglutinating, antimicrobial, antiaging, and anticancer activity. The main classes of compounds that have been extensively researched include erinacines (A-I) from *H. erinaceus* mycelium, and hericenones (C-H) from the fruit bodies [175]. A lot of research on this mushroom revealed that it has significant protective and anticancer effects in gastrointestinal malignancies. *H. erinaceus* has a protective effect on the chemically damaged mucus of the stomach and intestines, as well as proapoptotic and antimetastatic effects in colorectal cancer [176,177,178].

**Figure 5:**
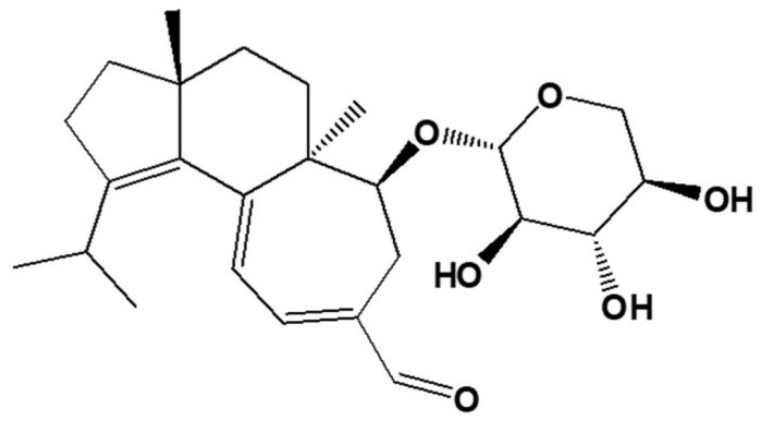
Erinacine A structure.

Gastric cancer, which ranks as the sixth most frequent in the world, is a malignant disease with poor prognosis [179]. Kuo et al. [180] analyzed the mechanisms of diterpenoid derivative erinacine A inhibition of gastric cell viability and invasiveness (Figure 5). *H. erinaceus* dried mycelium was extracted with 95% ethanol, and finally extraction of 5 mg/kg of *H. erinaceus* erinacine A with 85% ethanol was confirmed and quantified by HPLC. The exposure of human gastric cell line TSGH9201 to erinacine A led to the changes in cell morphology such as cell shrinking, rounding, and detachment that indicated cell toxicity. Flow cytometry indicated that the percentage of annexin positive cells was 13% (vs. 0.5% control) after 48 h. Matrigel assay revealed a significant inhibition in invasiveness and motility. Immunoblotting confirmed the induction of apoptosis based on the increase in TRAIL; cytochrome c; caspase-8, -9, and -3; and reduction of Bcl-2. Proteome expression was analyzed by 2DE SDS-PAGE. Reproducible changes in intensity of more than 2-fold were analyzed by MS/MS. Seventeen locations were subjected to protein identification by MALDI-TOF-MS. There were 17 proteins that were found to be differentially expressed, of which 4 were downregulated and 13 were upregulated. The protein microtubule-associated tumor suppressor candidate 2 (MTUS2), which plays a central role in controlling the microtubule and in cytoskeleton depolarization pathway, was upregulated. 14-3-3 protein sigma, which seems to be directly involved in human cancer through G2/M checkpoint control, was upregulated [181]. Based on the further experimental results, which confirmed ROS generation, as well as significantly increased total lysate protein with the phosphorylation (i.e., kinase induction) of FAK, AKT, p70S6K (ribosomal protein S6 kinase beta-1), and PAK1 after 6 h of erinacine treatment, it was concluded that erinacine exerts its inhibition on gastric cancer cell invasion and metastasis through ROS→p-FAK→p-AKT→p-p70mTOR→p-PAK→1433S/MTUS2 pathway [180]. Also, 14-3-3 protein zeta/delta, whose expression is elevated in multiple cancers and is correlated with poor prognosis based on its important roles in signal transduction, apoptosis, and cell migration, was found to be downregulated [181]. Nucleophosmin (B23), which has been found to be a negative prognostic marker in many types of cancer, including gastric cancer, was also downregulated [182]. This protein has important functions in ribosome biogenesis, genome stability and repair, and cell cycle [183].

Lee et al. [184] investigated erinacine A effects on the proteome of HCT-116 and DLD-1 colorectal cancer cells. It is known that levels of ROS are increased in colorectal cancer, so it is possible that phenolic phytochemicals that have antioxidant activity can modulate various ROS-dependent pathways and thus inhibit cancer cell proliferation [185]. Erinacine A was analyzed by HPLC. A scratch-wound assay demonstrated a significant inhibition in migration after 12 and 24 h, whereas treatment with 30 μM of erinacine A led to virtually complete inhibition of cell migration. Proteomic 2-DE analysis revealed 10 proteins for which the expression level was at least 3-fold greater in the erinacine A-treated group than in the untreated group. Nucleophosmin (NPM), known to be a negative prognostic marker in the clinic and elevated in many cancer, was downregulated. Hepatoma-derived growth factor (HDGF), which is involved in cellular proliferation, migration, invasion, and tumorigenesis of CRC, was likewise downregulated [186]. Profilin-1 and cofilin-1, which have important roles in erinacine A-induced ROS generation, were upregulated. Both are regarded as tumor-suppressor proteins, while cofilin-1 also has a role in controlling actin dynamics and actin depolymerization pathway, which controls the apoptosis and inhibition of cell migration in prostate, breast, and colon cancer cells through ROCK1/LIMK2/cofilin cascades [187,188]. Immunoblot analyses confirmed the upregulation of various tyrosine kinases, thus confirming that erinacine A produces elevated ROS and thereby activates ROS→p-PI3K→p-Akt→p-pmTOR→p-ROCK1/p-LIMK2→p-cofilin/profilin pathway, which results in inhibition of CRC metastasis. These results were also observed in a xenograft mice model, where intraperitoneal injections of erinacine A (1–5 mg/kg/day) significantly inhibited tumor volume and increased COFL1 (cofilin-1) and PROF1 (profilin-1) tissue protein expression. Furthermore, ROS scavenger NAC (N-acetylcysteine), PI3K inhibitor Wortmannin, and mTOR inhibitor rapamycin, as well as lentiviral control, shRNA COFL1, and shRNA PROF1, significantly reversed the erinacine A-induced cell death and cell migration. Therefore, it was concluded that *H. erinaceus* mycelium represent a novel chemotherapeutic agent worthy of continued investigation.

## 8. *Phellinus linteus*

*Phellinus linteus* (also known as mesima or black hoof mushroom) is a mushroom that mainly grows on mulberry tree trunks in the wild. Its various compounds, such as polysaccharides, proteoglycans, triterpenoids, phenylpropanoids, and furans, have been shown to exhibit immunomodulatory as well as direct antitumor effect [189]. Previous research demonstrated that novel proteoglycan P1 isolated from *Phellinus linteus* inhibits colorectal carcinoma by increasing the immune response of T cells and IgA and by disrupting the Reg IV/EGFR/Akt signaling pathway [190]. Li et al. [191] analyzed the antitumor mechanisms of *P. linteus* in hepatocellular carcinoma HepG2 cells. P1 was purified from *Phellinus linteus* fresh fruiting bodies and it was determined that its average molecular weight is approximately 18.8 kDa. Its polysaccharide composition was characterized and the protein content was 8.45%. The treatment of HepG2 cells with rising concentrations (50, 100, and 200 μg/mL) of P1 for 24, 48, and 72 h induced a concentration and time-dependent decrease in cell viability. IC50 in HepG2 after 48 h was about 125 μg/mL. Exposure of HepG2 cells to P1 (200 μg/mL) for 72 h increased the number of S-phase cell significantly, but the cell populations in G1 and G2/M phases were markedly decreased. This demonstrates that P1 induces S phase cell arrest. Since there was no statistically significant rise in either early or late apoptotic cells, and no evident sub-G0/G1 peak, this indicated that P1 inhibits HepG2 cell proliferation without apoptosis. Female nude Balb/c mice were inoculated with 0.2 mL of HepG2 (2.5 × 10^7^ cells/mL), and P1 was administered at doses of 100 and 200 mg/kg intragastrically every day. Cisplatin was used as a positive control and was injected at 2 mg/kg i.p. every other day. After 18 days, the volume and weight of tumors was significantly lower after treatment with 200 mg/kg P1 in comparison with the control group. Meanwhile, the mouse body weight did not show significant difference between P1-treated and control mice, indicating a lack of toxicity of P1 to mammals. In contrast, while cisplatin treatment resulted in a significant reduction in tumor volume, it also remarkably decreased the body weight of the HepG2-bearing mice, which suggested significant toxicity. For proteomic analysis, HepG2 cells were exposed for P1 (200 μg/mL) for 48 h, and 2-DE combined with LC-MS/MS analysis revealed several differentially expressed proteins, of which only one was significantly downregulated. MALDI-TOF/TOF revealed that it was a calreticulin (CRT) precursor. Calreticulin is a calcium-binding chaperone protein important in many cellular processes such as cell motility, cytoplasmic and mitochondrial metabolism, protein folding, cell cycle progression, and apoptosis [192]. It has been shown to be upregulated in breast cancer and to promote angiogenesis, proliferation, and migration in gastric cancer [193,194]. Also, this research demonstrated that the P1 inhibited HepG2 cell growth through induction of S phase arrest by activating the p27Kip1-cyclin A/D1/E-Cdk2 pathway.

## 9. Various Mushroom Genera

### 9.1. Grifola frondosa

*Grifola frondosa*, also known as maitake or hen-of the woods mushroom, has been used in Japan for many centuries. The best known compound from this mushroom is maitake D-fraction, which is a mixed β-d-glucan fraction of the fruiting bodies. It was shown that this fraction has direct antitumor (e.g., proapoptotic, antiangiogenic) as well as immunostimulating effects [195,196]. Unlike polysaccharides, which exhibit their antitumor effects primarily through immunostimulaton, glycoproteins are characterized by direct antitumor actions [197]. Cui et al. [198] analyzed the effects of *Grifola frondosa* glycoprotein GFG-3a in human gastric cancer cells. GFG-3a was obtained from the fermented mycelia and subsequently isolated and characterized. Treatment of human gastric cancer cell line SGC-7901 with increasing concentrations (10–200 μg/mL) of GFG-3a for 24 and 48 h resulted in significantly inhibited cell proliferation, which was dose- and time- dependent. Treatment of SGC-7901 cells with GFG-3a for 48 h resulted in dose-dependent cell apoptosis. Cell-cycle analysis demonstrated that GFG-3a caused cell cycle arrest at the S phase. Comparative proteomic analysis by 2-DE revealed that 21 proteins in the GFG-3a-treated groups were differentially regulated. Functional categorization revealed that the dysregulated proteins included cell cycle targets (RuvB-like 1, histone-binding protein RBBP4), stress response proteins (heat shock protein 90-beta—HSP90B, 78 kDa glucose-regulated protein—GRP78), nucleic acid-related function (nucleophosmin, heterogeneous nuclear ribonucleoprotein F), and cytoskeleton (tubulin, keratin). RuvBL1 protein is known to be upregulated in gastric, colorectal, and other cancers, and is essential in cell replication, so its downregulation by GFG-3a could induce S phase arrest and p53 activation [198,199,200]. Downregulation of nucleophosmin, which has been proposed as a tumor marker for gastric, colon, ovarian and prostate cancers, could induce apoptosis and cell cycle arrest [201]. HSP90B and GRP78 are molecular chaperone proteins expressed in the ER and are included in protein structural maintenance. Stress response proteins are induced in response to many environmental stimuli including heat, ROS, and cytotoxic drugs [202]. The downregulation of these proteins by GFG-3a may contribute to p53-regulated cell apoptosis [198]. Additionally, this study has shown that GFG-3a treatment significantly upregulated proapoptotic proteins caspase-3 and -8, p53, and Bad, while Bcl-2, PI3K, and Akt were downregulated.

### 9.2. Antrodia cinnamomea

Chen et al. [203] examined the effect of *Antrodia cinnamomea* on the differential proteomic patterns in liver cancer cell lines HepG2 and C3A, and Chang’s liver cell, a normal liver cell line, by using a quantitative proteomic approach. Besides various antitumor effects, this fungus has been demonstrated to have liver-protective activity, through its inhibition in a liver injury rat model [204,205,206]. Fluorescently-label technique 2D-DIGE was used, since it has a broader dynamic range of protein detection, higher detection sensitivity, and greater reproducibility than regular 2-DE [207]. *A. cinnamomea* extract was obtained from the fruiting body by 95% ethanol. The content of the main active components, triterpenoids, was 9.4%. Cell viability assay demonstrated that *A. cinnamomea* exhibited a cytotoxic effect in a concentration range of 0–1000 μg/mL, with a significant decrease of cell viability (50%) at 400 μg/mL and further. Since non-tumorigenic Chang’s liver cells had a higher IC50 value at this concentration than hepatocellular carcinoma C3A and HepG2 cells, this implied that ethanolic extract of *A. cinnamomea* (EEAC) was less toxic to non-tumorigenic cells, i.e., that the effect was specific. Treatment with EEAC caused a significant induction of mostly late apoptosis, which was much more pronounced in liver cancer cells C3A (29.7%) and HepG2 (57.6%), than in non-tumorigenic Chang’s liver cells (5.7%). For the proteomic analysis, C3A, HepG2, and Chang’s liver cells were treated with 400 μg/mL of EEAC or vehicle. 2D-DIGE/MALDI-TOF MS-based proteomic strategy was used. DIGE revealed more than 1500 defined protein features, and those with >1.2 fold change (*p* < 0.05) were excised. Mass spectrometry identification demonstrated 82, 125, and 125 differentially expressed proteins between *Antrodia cinnamomea*-treated and untreated Chang’s liver cells, C3A cells, and HepG2 cells, respectively. In general, protein abundance comparison showed that the proteins known to modulate protein folding, redox-regulation, glycolysis, and transcription control were downregulated in both C3A cells and HepG2 cells compared to normal Chang’s liver cells. In contrast, proteins known to regulate cytoskeleton regulation and signal transduction were found to be upregulated in both C3A and HepG2 cells than that in Chang’s liver cells. The identified proteins were imported into STRING database, and protein–protein interaction analysis indicated that the dominant functional alterations in Chang’s liver cells were apoptosis and cytoskeleton regulation and in C3A and HepG2 cells stress response and redox regulation. Namely, dysregulation of cellular protein folding (indicated by dysregulation of PDIA3; HSPB1; HSPA8; HSP5; Erp29; glucose-regulated protein 78, GRP78; eIF2-alpha; IRE1-α; XBP1; ATF6; GADD153; calnexin; and ERO1L) in ER, which leads to apoptosis, is one of the primary mechanisms that are thought to be involved in liver cancer cell death. It was also found that dysregulation of proteins involved in intracellular redox status resulted in ER stress. Proteomic analysis demonstrated a downregulation of mRNA processing proteins and heterogeneous nuclear ribonucleoproteins A2/B1 and K, which has be shown to induce TRAIL-mediated apoptosis [208]. Importantly, EEAC treatment induced downregulation of glycolytic enzymes (PKM1, PGAM1, LDHA, GAPDH, G6PD, ENO1, and ALDOA), which might result from *A. cinnamomea* effect on Bad upregulation (proapoptotic protein) and the elevated oxidative stress that shunts metabolism to NADPH production [209,210].

### 9.3. Auricularia auricula

This mushroom, also known as black fungus or tree ear, has mainly been studied in the context of its hypoglycemic and hypocholesterolemic effects [211]. Kang et al. [212] investigated the antitumor mechanisms of this fungus in hepatocellular carcinoma cells. Three types of *A. auricula* isolates were used: polysaccharides obtained by freeze-drying (FD), microbe (Baekdu No.1) bioconverted *A. auricula* extract supernatant dissolved in PBS (BS), and BT (bioconverted *A. auricula* total extract solution dissolved in PBS). Among the five hepatocellular cancer cell lines (HCT-15, huh-7, SK-MEL-5, SNU-213, and SNU-484), *A. auricula* extracts showed the most significant viability decrease in huh-7 cells at 0–1 mg/mL, which were therefore chosen for subsequent experiments (BS IC50 on Huh-7 cells was 0.8 mg/mL after 24 h). In Huh-7 cells, BS was most effective in increasing apoptosis. Since BS was most effective at antiproliferation and apoptosis, it was used for proteomic analysis. 2DE-GE detected seven protein spots and they were analyzed by MALDI-TOF/MS. Six proteins were upregulated (leucine-rich repeats and immunoglobulin-like domains 3, ATP synthase subunit d, ubiquitin-conjugating enzyme E2 K, keratin, type I cytoskeletal 9, human homolog of Mus musculus wizL protein, aldo-keto reductase family 1 member C3), while peroxiredoxin-1 (PRDX1) was downregulated. Research was further focused on PRDX1, which is an important antioxidant enzyme. It was hypothesized that the downregulation of PRDX1 in cancer cells reduces their ability to deal with the elevated ROS levels, which induces cell death. PRDX1 was shown to enhance cell proliferation, protect against ROS, and is involved in resistance against radiotherapy [213]. Using PRDX1 siRNA in Huh-7 cells, a decrease in GSH was detected. GSH is an endogenous antioxidant that enables antioxidant defense, so its downregulation increases ROS, which leads to apoptosis [214]. Also in PRDX1 siRNA and BS-treated groups, a decrease in SOD was detected, which points to the inactivation of the antioxidant enzymes in treated cells. *A. auricula* treatment (FD, BT and BS) induced an upregulation of proapototic Bak; Bid; Bik; PARP; and cleaved caspases-3, -8, and -9, while it downregulated Akt and Bcl-xL.

### 9.4. Sporisorium reilianum

*Sporisorium reilianum* is a kind of biotrophic fungal plant pathogen that causes sorghum head smut. Kan et al. [215] investigated the antitumor properties of *S. reilianum* polysaccharide WM-NP-60 on colorectal adenocarcinoma HCT-116 cells. This polysaccharide is water-soluble and has a molecular weight of 15.6 kDa. It was previously established that this polysaccharide has good antitumor activity against hepatocellular carcinoma HepG2 and SCG7901 cells [216]. WM-NP-60 at a concentration of 8mg/mL could reduce cell viability by nearly 40%. WM-NP-60 treatment increased G1 cell population, and this suggested that it can cause G1 cell arrest. WM-NP-60 also increased the number of apoptotic HCT-116 cells compared with the control group. For proteomics, HCT116 cells were treated with 4 mg/mL for 48 h. After BCA quantification and SDS-PAGE electrophoresis, the samples were TMT-labeled. From 7355 proteins, 369 significantly differentially expressed proteins (FC > 2 or FC < 0.5, *p* < 0.05) were identified, of which 240 were upregulated and 129 were down-regulated. GO enrichment analysis of significantly differentially expressed proteins showed that the top differentially expressed proteins by biological process were: secretion by cell and the exocytosis, platelet degranulation, protein activation cascade, regulation of protein maturation, and regulation of protein processing. KEGG analysis (pathway enrichment) revealed that significantly differentially expressed proteins could be categorized into five specific pathways involved in metabolism, cellular processes, environmental information processing, organismal systems, and human diseases. Some of the upregulated proteins found to be differentially expressed such as TGFβR1 (transforming growth factor beta receptor I), P107, DP1 (transcription factor Dp-1), ITGA7 (integrin alpha 7), and Rap1 (Rap1 GTPase-activating protein 1) are important in various signaling pathways and processes, such as TGFβ, Hippo, MAPK, PI3K-Akt, Ras and chemokine signaling pathways, cell cycle, cell adhesion etc. The downregulated THBS1 (thrombospondin 1) was associated with p53, PI3K-Akt, Rap1, and TGF-β signaling pathways, as well as focal adhesions and ECM–receptor interaction. By using the STRING database, protein–protein interactions (PPI) were confirmed between some of these proteins: between upregulated TGFβR1, P107, and DP1, and downregulated THBS1. These proteins are in the TGF-β signaling pathway, which is related to cell cycle and apoptosis. Proteomic results were confirmed by Western blot and qRT-PCR analysis. It was concluded that the antitumor mechanism of WM-NP-60 might be connected to the changed expression of TGFβR1, P107, DP1, ITGA7, Rap1, and THBS1 in HCT116 cells. Decreased THBS1 is related to malignant progression and reduced survival of cancer cells, so it was concluded that its downregulation might promote apoptosis and inhibit colon cancer invasion and metastasis [216,217]. TGFβ is a growth factor that prevents progress from G1 to S phase and inhibits cell proliferation; therefore, the upregulation of TGFβR1 by WM-NP-60 was thought to have promoted the arrest of HCT-116 cells at G1 phase and induced apoptosis. ITGA7, a primary laminin receptor that is involved in maintenance of the myofibers cytoarchitecture, anchorage, and viability, was found to be able to inhibit the proliferation and metastasis of CRC cells by inhibiting Ras [218]. Thus, one of the mechanisms of WM-NP-60 in blocking the cell cycle at G1 phase is by upregulating ITGA1 and Rap1 proteins in a focal adhesion pathway.

### 9.5. Agrocybe aegerita

*Cyclocybe aegerita*, also called *Agrocybe aegerita* and commonly known as poplar or chestnut mushroom, is a high quality edible mushroom that is cultivated all over the world. Wang et al. [219] studied the effects of fraction 2 proteins of *Agrocybe aegerita* (AA-f2-MNC-CM) on U937 lymphoma cells. It was found previously that the proliferation of human leukemic U937 cells was reduced by about 80% when the cells were incubated with conditioned media (CM) of human blood mononuclear cells (MNC) stimulated with 100 mg/mL of cold water extracts (CWE) proteins from *Hypsizigus marmoreus* or *Agrocybe aegerita* [220]. Two protein fractions (AA-f2) were obtained from the *A. aegerita* fruiting bodies. Mononuclear cell-conditioned medium (MNC-CM) was prepared by incubating the cells in the presence of 5 or 25 μg/mL of AA-f2 for 24 h. Conditioned media collected from AA-f2-treated MNC cultures was designated as AA-f2-MNC-CM. Human myeloid leukemic U937 cells were maintained at an initial concentration of 1 × 10^5^ mL of AA-f2-MNC-CM or PBS-MNC-CM for 5 days. Protein separation was conducted by 2-DE. It was found that 87 protein spots in the 5 μg AA-f2/mL and 54 protein spots in 25 μg AA-f2/mL were relatively different in quantity (≥50%), and these spots were identified as MALDI-TOF MS. In both groups, all identified proteins were downregulated after AA-f2-MNC-CM treatment. Among the 53 individual proteins that were successfully identified, 7 were downregulated in both groups (heterogeneous nuclear ribonucleoprotein, macrophage capping protein, pyruvate dehydrogenase E1 component beta subunit mitochondrial precursor, glutathione transferase omega 1, pyruvate kinase M1 isozyme, endoplasmic reticulum protein Erp29 precursor, and glyceraldehyde 3-phosphate dehydrogenase). Fifty-three proteins that were detected to be downregulated had the following biological functions: synthesis of DNA and RNA, catabolism pathways (such as glycolysis pathway, pentose phosphate pathway, citric acid cycle, and uric acid synthesis) and protein conformation. So, it was concluded that the inhibition of proliferation and differentiation of U937 cells by AA-f2-MNC-CM is associated with the inhibition of the disulfide bond formation and proteolysis of cellular proteins, interruption of catabolism pathways, accumulation of toxic intermediates or toxic substance, and replication inhibition of DNA or RNA that inhibits the U937 biosynthesis [219].

### 9.6. Nectria haematococca

This fungus from the Ascomycota division is a common soil fungus and plant as well as a human pathogen [221]. Xie et al. [222] investigated its properties as an antitumor agent on human lung adenocarcinoma. Fungal immunomodulatory proteins (FIPs), which are widely found in fungi, have strong inhibitory effects on lung carcinoma. More than 10 FIPs have been isolated and identified, of which the most well-known are FIP from *Ganoderma lucidum* (LZ-8 or FIP-glu), *Ganoderma tsugae* (FIP-gts), *Flammulina velutipes* (FIP-fve), and *Nectria hematococca* (FIP-nha) [223,224,225]. FIP-nha, which was the subject of this research, consists of 114 amino acid residues, and has a molecular weight of 12,837 Da. A previous study demonstrated that FIP-nha strongly suppresses the growth of A549 lung adenocarcinoma cells, with an efficacy superior to that of FIP-fve or LZ-8 [222]. In this research, cell viability of human lung adenocarcinoma cell lines A549 and H2347 (NCI-H2347) treated with FIP-nha in a concentration of 0–20 μg/mL for 24 h exhibited a dose-dependent decrease in viability up to 80%. In contrast, only very slight cytotoxicity was observed in healthy lung MRC-5 fibroblasts, indicating a selective antitumor effect. In vivo research utilized Balb/c nude mice in which the tumor xenografts were established by subcutaneous injection of 5 × 10^6^ A549 cells, and treated with PBS (control), FIP-nha (20 mg/kg), FIP-nha (40 mg/kg), or doxorubicin (4 mg/kg) every week. The average tumor volume in the high dose (40 mg/kg) FIP-nha was significantly decreased in comparison to the negative control group at day 33, with the efficacy in decreasing tumor volume equivalent to that of the chemotherapy drug doxorubicin. Global proteomic analysis was done by isobaric tags for relative and absolute quantification (iTRAQ)-based quantitative proteomic analysis on A549 cells treated with 8μg/mL FIP-nha for 24 h. Significantly (*p* ≤ 0.05) differentially regulated proteins were those with a fold change of ≥1.2 or ≤0.83. Out of 2650 proteins identified, 334 differentially expressed proteins fit those criteria, with 213 being upregulated and 121 downregulated. Functional analysis (enrichment significance) confirmed that the upregulated proteins were mainly involved in extracellular matrix organization, PI3K/Akt signaling, cell apoptosis, cell autophagy, and cell migration, while downregulated proteins were mainly enriched in functions including G1/S and G2/M cell cycle arrest, ubiquitination, telomere maintenance, and cell proliferation. Further analysis (Western blot) confirmed that FIP-nha induced a dose-dependent (0–16 μg/mL) decrease in the expression of both Akt (p-Akt) as well as mTOR (p-mTOR), thus confirming the critical role of PI3K/Akt pathway in A549 and H2347 lung adenocarcinoma cells. The heightened expression of LC3B-II in a dose-dependent manner indicated the induction of autophagy. Cell cycle analysis and Western blot indicated that FIP-nha treatment results in G1/S cycle arrest and G2/M phase transition. Flow cytometry showed a significant apoptosis induction in A549 (from 5.70% to 52.95%) and H2347 (from 10.56% to 59.11%) cells. Although the induction of autophagy by FIP-nh had a protective effect on tumor cells, it was confirmed previously that some FIPs, such as FIP-gmi, induced cell death through activating autophagy in A549 cells [226]. The in vivo results in a nude xenograft mouse model confirmed that FIP-nha inhibits lung adenocarcinoma cell growth through regulating cell cycle arrest, autophagy, and apoptosis. Although FIP was given intraperitoneally, it is known that FIPs are highly resistant to acid hydrolysis, alkali decomposition, and enzyme digestion, which means that they can be administered orally as well [227].

### 9.7. Agaricus bisporus

*Agaricus bisporus*, also known as common mushroom, button mushroom, champignon mushroom, or portobello mushroom, is one of the most commonly and widely consumed mushrooms in the world. Mahmood et al. [228] studied the antitumor effects of a lectin isolated from *A. bisporus* (ABL—*Agaricus bisporus* lectin) conjugated with CaCO_3_ nanoparticles (ABL- CaCO_3_NPs) on MCF-7 breast cancer cells. Fungal lectins are known to have significant pharmacological potential, which includes antioxidant, antitumor, and immunostimulating properties [229]. Nanotechnology has enabled new possibilities of drug delivery, which can increase drug efficiency of traditional chemo- and radiotherapies by focusing on the target site [230]. For cell cycle analysis, MCF-7 cells were treated with 75 μg/mL (IC50 concentration) of ABL-CaCO_3_NPs or with 4 μg/mL of doxorubicin as positive control for 24 h. ABL-CaCO_3_NPs treatment caused a significant increase in cells in G1 phase compared with both untreated and doxorubicin-treated cells. Cell cycle arrest, which is also a mechanism reported for many chemotherapeutic agents, leads to apoptosis [231]. After treating MCF-7 cells with the same concentrations of either ABL-CaCO_3_NPs or doxorubicin (DOX) for 24 h, gel-based 2-DE proteomic analysis revealed 22 statistically significant (*p* ≤ 0.05 and fold change ≥ 2) differentially abundant spots, which were further analyzed. LC-MS/MS identified 13 proteins that were dysregulated. Treatment with ABL-CaCO_3_NPs downregulated V-set and immunoglobulin domain, serum albumin, actin cytoplasmic 1, triosephosphate isomerase, tropomyosin alpha-4 chain, and endoplasmic reticulum chaperone BiP in comparison with both negative and positive control (DOX); while hornerin, tropomyosin alpha-1 chain, annexin A2, and protein disulfide-isomerase were upregulated in comparison to the positive control and downregulated in comparison to the negative control. Ingenuity pathway analysis (IPA) revealed that the upregulated proteins hornerin (HRNR), tropomyosin alpha-1 chain (TPM1), annexin A (ANXA2), and protein-disulfide isomerase (PDI) were associated with breast or gynecological cancer, breast carcinoma, and inflammation of organ. Breast cancer-related protein hornerin (HRNR), part of S100 protein family that is involved in malignant transformation, cell proliferation, differentiation, and death, and known to be elevated in some cancers, was not significantly altered in the ABL-CaCO_3_NPs group, but was increased in DOX group, which could be an indicator of poor prognosis [228,232,233]. From the molecular chaperones/heat shock protein group, protein disulfide isomerase (PDI) was downregulated in ABL-CaCO_3_NPs-treated group with respect to negative control and upregulated in comparison with DOX. PDI is known to be involved in the unfolded protein response (UPR) pathway and is correlated with worse outcome in breast and other carcinomas, so its downregulation could enhance apoptosis in MCF-7 cells [228,234,235]. Binding immunoglobulin protein (BiP), also known as glucose-regulated protein 78 (GRP78), is a protein from the same molecular chaperone group. BiP was found to be downregulated as a result of ABL-CaCO_3_NPs treatment in comparison with both controls. Increased BiP, which regulates UPR signaling, is known to improve cancer cell proliferation, apoptosis resistance, immune evasion, metastasis, microenvironmental angiogenesis, and therapy resistance [236]. From the cytoskeleton and associated proteins group, both actin citoplasmic 1 or beta-actin (ACTB) and tropomyosin alpha 1 chain and alpha-4 chain (TPM1 and TPM4) were downregulated. Abnormal abundance of ACTB is associated with cancer invasiveness and metastasis, and its high abundance in DOX-treated cells and low abundance in ABL-CaCO_3_NPs can potentially inhibit Erk1/2 and decrease cycline A and decelerate proliferation and anti-apoptotic responses [228]. TPM1 and TPM4, which were downregulated in comparison with both controls, are known to be differentially abundant in breast cancer epithelial vs. normal breast tissues [237]. From the metabolic enzymes group, triosephosphate isomerase (TPI), a glycolysis pathway enzyme, was shown to be downregulated in ABL-CaCO_3_NPs group compared with both controls and is thought to be related with a decrease in energy requirements in treated cells. A group of membrane-associated proteins with multiple activities including annexin A2 (ANXA2), human serum albumin (ALB), and V-set and immunoglobulin domain (VSIG-8), were all found to be downregulated as a result of ABL-CaCO_3_NPs treatment. Treatment with ABL-CaCO_3_NPs resulted in downregulation of ANXA2 with respect to negative control and upregulation in comparison with DOX. ANXA2 has been reported to be involved in breast cancer neoangiogenesis, invasiveness, and resistance [238]. ALB, the most abundant protein in serum, has been proposed to be used by tumors as an energy source and was shown to accumulate in solid tumors [239]. VSIG-8 was found to be a promising immune checkpoint molecule capable of inhibiting human T cell activation [240]. Bioinformatic analysis showed that the dysregulated proteins were involved with various pathways associated with breast cancer. Upregulated ANXA2 was associated with the inhibited BAG2 signaling pathway, which is usually highly abundant in breast cancer and promotes tumor progression and metastasis [241]. Low abundance of ACTB and ALB proteins are associated with caveolar-mediated and clathrin-mediated endocytosis signaling. These pathways are relevant in increased invasion and metastasis, so ABL-CaCO_3_NPs treatment might impair the caveolar-mediated endocytosis, resulting in an antitumor effect. The upregulation of PDI affected the pathways of UPR, hypoxia, and the role of tissue factor in cancer. Low abundance in ACTB and GRP78 affected EIF2 signaling and ER stress. PDI upregulation and therefore UPR are correlated with elevation of hypoxic state and triggering of ER stress in tumor cells to reestablish intracellular homeostasis and promote cell survival [234]. Downregulated TPM1 and TPM4 were involved in the calcium signaling pathway, which is involved in calcium-binding S100A4 protein implicated in cancer metastasis. Therefore, the low abundance of these TPMs would most likely affect the migration of breast cells [242].

## 10. Ergosterol

**Figure 6:**
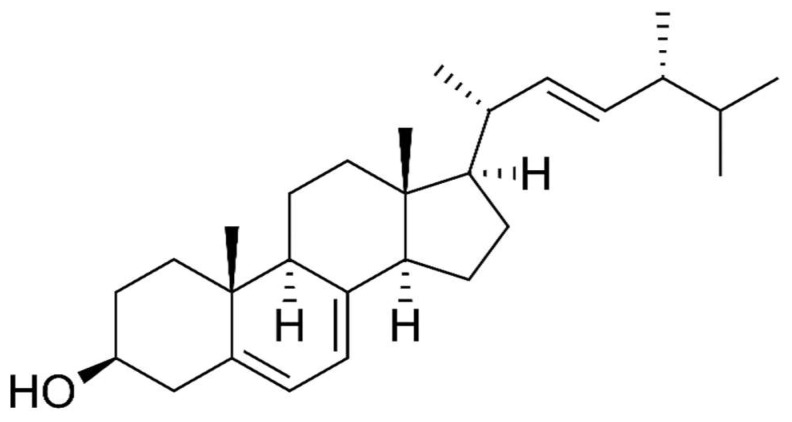
Ergosterol structure.

Ergosterol is the principal sterol in fungi (and pro-vitamin D_2_ in human nutrition), where it represents about 70% of all sterols (Figure 6). Besides in mushroom cell membranes, it is also present in protozoa [243]. Kuo et al. [244] analyzed the proteomic response in RAW 264.7 macrophages treated with ergosterol in studying its anti-inflammatory properties. It is known that ergosterol peroxide suppresses the LPS-induced inflammatory responses through inhibition of NF-κB transcriptional activity and phosphorylation of MAPKs [245]. Cytotoxicity of ergosterol in concentrations of 1–100 μg/mL on LPS-stimulated mouse RAW 264.7 macrophages were tested. Ergosterol showed a dose-dependent cytotoxic effect with an IC50 of 24.5 μg/mL. In order to rule out cytotoxic effects of high-concentration treatments, ergosterol was only used in a concentration range of 0–20 μg/mL in subsequent experiments. The flow cytometry showed that there was no apoptotic effect of ergosterol on RAW 264.7 cells, which indicated that the inhibition of proliferation was not due to increased apoptosis. In LPS-activated macrophages, a significant increase in both NO and TNF-α was observed. It was shown that that the exposure of LPS stimulated RAW 264.7 cells to rising concentrations (0–20 μg/mL) of ergosterol, which induced a slight reduction of NO generation and a significant, dose-dependent suppression of TNF-α production. Western blot showed that treatment of the cells with 10 μg/mL decreased LPS-induced COX-2 expression but did not affect iNOS expression. The proteome of LPS-induced RAW 264.7 cells treated with 10 μg/mL ergosterol was analyzed by 2-DE. Protein spots were subjected to MALDI-TOF/TOF mass spectral measurements and PMF/MS-MS. The proteins upregulated by ergosterol treatment included plasminogen activator inhibitor 2 (PAI2), cytoplasmic actin 1, vimentin, and tubulin α-1C chain. The proteins downregulated by ergosterol treatment were Rho GDP-dissociation inhibitor 1 (Rho GDIS1), ataxin-3, T-complex protein 1 subunit theta (TCP1θ), and TRAF family member-associated NF-κB activator (TANK). The downregulation of NF-κB-associated protein, TANK, and Rho GDIS1 (chaperone protein) indicate that ergosterol may have inhibited NF-κB activation by regulating upstream IκB through Rho GTPase modulation of IKK. Upregulated proteins involved in cytoskeleton structure and function included cytoplasmic actin 1, vimentin, and tubulin α-1C chain. Chaperone proteins, including TCP1θ, HSP90, and HSP60, are implicated in reorganization and folding of cytoskeleton proteins, but Western blot analysis showed that HSP90 and HSP60 were not significantly altered after treatment with ergosterol, which indicated that these proteins did not contribute to the change of cytoskeleton-related proteins [244,246].

## 11. Medicinal Mushroom Mixtures

Several studies have shown that combining multiple medicinal mushroom species with known antitumor effects may have additive or synergistic effects in terms of immune system activation [247,248,249]. It is known that various polysaccharides may activate multiple receptors on immune cells, and mushroom mixtures may also exhibit an additive affect in activating various direct anticancer mechanisms such as interfering with multiple pathways relevant in tumor growth and progression [13,250]. Jakopovic et al. [251] analyzed the proteome profile of tumor tissues of mice treated with medicinal mushroom extract mixture in a colorectal cancer model. Agarikon.1 preparation, a medicinal mushroom extract mixture, is a registered dietary supplement that contains a mixture of *Lentinus edodes*, *Ganoderma lucidum*, *Agaricus brasiliensis* (=*blazei* ss. Heinem.), *Grifola frondosa*, *Pleurotus ostreatus*, and *Trametes versicolor* in equal amounts i.e., 125 mg per 1000 mg tablet [249]. One tablet therefore contains 750 of mushroom polysaccharides and excipients such as inulin, talc, magnesium stearate, and silica. This tablet preparation is produced by water extraction followed by ethanol precipitation and subsequent freeze-drying. Balb/c mice were injected with 1 × 10^6^ CT26.WT cells. The 14 day treatment with Agarikon.1 (1200 mg/kg i.g.) or 5-fluorouracil as a positive control (30 mg/kg on days 1.–4. and 15 mg/kg on 6., 8., 10., and 12. day of treatment) started only when tumors were developed in 100% of the animals and when the tumor size was ≥700 mm^3^ i.e., 14 days post tumor implantation, which represents a very advanced, grade IV tumor model. The third group received both 5-fluorouracil (5-FU) and Agarikon.1 (AG.1) in aforementioned concentrations, and the fourth, 5-FU only. Doses were calculated by allometric scaling [252]. The survival analysis that was conducted until day 55 demonstrated that survival rates were 50% with AG.1, 77.7% with 5-FU, and 33.3% in AG.1 + 5-FU group, which indicates that there was no additive effect in terms of survival in the combination group. In the preventive group, where the same doses of AG.1 were administered for a week before and after tumor inoculation, 100% of mice were still alive up to day 45 post tumor inoculation. Tumor volume was significantly reduced only in 5-FU in comparison with control. In cancer clinical trials, overall survival is considered a definitive end point [253]. It has been established that the volume of the primary tumor has no prognostic significance in CRC, which is also valid for the preclinic in CT26.WT CRC model [254,255]. AG.1 was previously shown to significantly inhibit viability and induce apoptosis in HCT-116 and SW620 human adenocarcinoma cells in vitro, as well as induce macrophage polarization and inhibit tumor angiogenesis in vivo [256]. Tandem mass tag (TMT) proteomic analysis of tumor tissue revealed a total of 95 dysregulated proteins, of which 36 were upregulated and 59 were downregulated. Bioinformatic analysis of the upregulated and downregulated proteins in each group was performed by STRING (protein-protein interactions) and REACTOME (pathway enrichment). It was demonstrated that for the AG.1 and AG.1 + 5-FU group, downregulated proteins were involved in ribosome assembly, translation, and ribosome biogenesis-enriched pathways (40S ribosomal protein SA–Rpsa, ribosome maturation protein SBDS–Sbds, 40S ribosomal protein S3–Rps3, eukaryotic initiation factor 4A-I and II–Eif4a1 and Eif4a2 etc.). It has been shown that global dysregulation of translation, particularly its progressive upregulation, is a key driver in cancer pathogenesis [257]. In the 5-FU only treated group, the upregulated proteins (60S ribosomal protein L29–Rpl29) were involved in an enriched translation pathway, which may indicate a 5-FU rapid effect on translation machinery, activated 5-FU extraribosomal functions, or 5-FU resistance [258]. Further PPI and pathway analysis revealed that downregulated proteins in AG.1 and AG.1 + 5-FU groups (Protein S100-A9, Annexin A5 in AG.1 group, heterogeneous nuclear ribonucleoproteins in AG.1 + 5-FU group) participated in enriched mRNA splicing and mRNA processing pathways, and influenza life cycle pathway, which are the three most relevant pathways that are gradually upregulated during CRC progression [259]. Upregulated proteins in all treated groups were involved in unfolded protein response (UPR), which was indicated by the DnaJ homolog subfamily C member 3 (Dnajc3) and its interactors up-accumulation. It was reported that activation of UPR causes differentiation in colon cancer stem cells, which enhances their responses to chemotherapy in vitro and in vivo [260]. By analyzing the temporal dynamics of differentially upregulated proteins during CRC progression, Peng et al. showed that the UPR was mostly downregulated during CRC progression [259]. Furthermore, enrichment analysis showed that various upregulated proteins in AG.1, AG.1 + 5-FU, and 5-FU (apolipoprotein A-II Apoa2, cytosolic acyl coenzyme A thioester hydrolase–Acot7) were involved in regulation of lipid metabolism and TCA cycle pathways (fumarate hydratase, mitochondrial, Fh). These are important in promoting fatty acid oxidation, which has proven to be downregulated in multiple tumors, as well as in reprogramming cancer metabolism from pentose phosphate pathway (PPP) back to increased tricarboxylic acid cycle (TCA), thus reversing Warburg effect, respectively [261,262]. Thus, it was concluded that AG.1 alone, or in combination with 5-FU, evoke changes contrary to those found during progression of colorectal carcinogenesis, which results in significantly improved survival.

## 12. Conclusions

Natural products are an effective source of anti-tumor agents, and it is estimated that more than 80% of anti-cancer drugs are either natural products or are based on them [263]. The main goal of functional proteomics and bioinformatic analysis in cancer medicine is to detect potential pathways and protein targets that can be exploited by various pharmacological approaches. In this regard, it could be viewed as a complimentary approach to network pharmacology, a new systems biology discipline. Network pharmacology has also begun to be used in the medicinal mushroom field [264,265,266]. The complexity of cancer, which includes various pathways of its ontogeny and progression, tumor microenvironment, and therapeutic resistance mechanisms, poses demanding challenges that require a systems biology approach that is now increasingly beginning to re-examine the historical reductionist approach. In this regard, the previous research on cancer as well as on cancer therapeutics might be regarded as preliminary or partial and in need of a more in-depth study. However, various challenges remain. Besides the complexity and variability of biological material, i.e., medicinal mushrooms, the variability in the results may also be influenced by the model, either in vitro or in vivo, where the timing of the treatment (early vs. late models of disease; immunocompetent vs. nude mice xenografts), as well as the tumor model (orthotopic vs. heterotopic), can produce different results. The results are also highly dependent on the proteomic methods, which also differ in their sensitivity. However, these obstacles must be overcome in order to study complex diseases such as cancer.

## Figures and Tables

**Table 1:** Primary research included in this review and its experimental parameters.

Mushroom Species	Compound	Type of Experiment (Tumor Model)	Proteomic Methodology	Validation	Reference
*Ganoderma lucidum*	Characterized *Ganoderma lucidum* spores (GL-SP)	In vitro Splenic mononuclear cells (MNCs)	2-DE followed by MALDI-MS/MS	No	Ma, C., et al., 2008
*Ganoderma lucidum*	Characterized *Ganoderma lucidum* spores (GL-SP)	In vivo Thymus lymphoid cells	2-DE followed by MALDI-TOF MS/MS	Western blotting	Ma, C., et al., 2009
*Ganoderma lucidum*	Characterized *Ganoderma lucidum* polysaccharide peptide (*Gl*PS) from the fruiting body	In vivo Murine sarcoma S180 model	2-DE followed by ESI-Q-TOF-MS/MS	Western blotting and RT-PCR	Li, Y.B., et al., 2008
*Ganoderma lucidum*	Characterized Ganoderma lucidum polysaccharide peptide (GL-pp)	In vivo Murine melanoma (B16-F10-luc-G5) model	LC-MS/MS followed by bioinformatic analysis	No	Xian, H., et al., 2021
*Ganoderma lucidum*, *Phellinus linteus*, *Auricularia auricula*	Polysaccharides from *Ganoderma lucidum* (GL), *Phellinus linteus* (PL), *Auricularia auricula* (AA) studied separately	In vitro Human HepG2 (hepatocellular carcinoma) cell line	2-DE followed by MALDI-TOF-MS and bioinformatic analysis	Western blotting and RT-PCR	Chai, Y., et al., 2016
*Ganoderma lucidum*	Fungal immunomodulatory protein Ling Zhi-8 (LZ-8)	In vivo Murine Lewis lung carcinoma cell line (LLC1)	2-DE followed by LC-MS/MS and bioinformatic analysis	Western blotting	Lin, T.Y., et al., 2021
*Ganoderma leucocontextum*	GL22 triterpene	Huh7.5 liver cancer cell line in vitro and in vivo (xenograft) mouse model	LC-MS/MS followed by bioinformatic analysis	Western blotting	Liu, G., et al., 2018
*Ganoderma lucidum*	Ganoderic acid D	In vitro HeLa human cervical carcinoma cells	2-DE followed by MALDI-TOF MS/MS	Western blotting	Yue, Q.X., et al., 2008
*Ganoderma lucidum*	ganoderic acid F (GAF), ganoderic acid K (GAK), ganoderic acid B (GAB), ganoderic acid D (GAD) and ganoderic acid AM1 (GAAM1)	In vitro HeLa human cervical carcinoma cells	2-DE followed by MALDI-TOF MS/MS	Western blotting	Yue, Q.X., et al., 2010
*Ganoderma lucidum*	Characterized mixture of *Ganoderma lucidum* triterpenes (GTS)	In vitro HeLa human cervical carcinoma cells	2-DE followed by MALDI-TOF MS/MS	Western blotting	Yue, Q.X., et al., 2008
*Lentinus edodes*	MPSSS polysaccharide	In vitro Prostate CAFs (cancer associated fibroblasts) and PC-3 (human prostate cell line)	Tandem mass tag (TMT) labeling followed by LC-MS/MS and bioinformatic analysis	Western blotting	Zhang, T., et al., 2021
*Lentinus edodes*	Lentinan	In vivo Murine H22 hepatoma cells	LC-MS/MS	No	Wang, Y., et al., 2017
*Lentinus edodes*	Lentinan	In vivo ascites and solid H22 liver cancer models	LC-MS/MS	No	Yang, X., et al., 2020
*Lentinus edodes*	Lentinan	In vivo Murine H22 hepatoma cells	HPLC-MS/nMS	ELISA	Wang, W., et al., 2021
*Lentinus edodes*	Lentinan (LNT)-functionalized selenium nanoparticles	In vivo Ovarian cancer (OVCAR-3) and Erlich ascites carcinoma (EAC) models	LC-MS/MS followed by bioinformatic analysis	Western blotting	Liu, H.J., et al., 2020
*Cordyceps militaris*	*Cordyceps militaris* (CM) fresh body or mycelia extract	In vitro Cisplatin-resistant A549 human lung adenocarcinoma cells	Protein antibody microarray	Western blotting	Jeong, M.K., et al., 2019
*Cordyceps sinensis*	Characterized *Cordyceps sinensis* water extract (WECS)	In vitro and in vivo 4T1 breast cancer model	Protein array	No	Cai, H., et al., 2018
*Cordyceps sinensis*	Characterized *Cordyceps sinensis* ethanol extract	In vivo DEN (diethylnitrosamine)-induced hepatocellular carcinoma	2-DE followed by MALDI-TOF MS and bioinformatic analysis	Western blotting	Wang, P.W., et al., 2016
*Cordyceps cicadae*	Water extract of *Cordyceps cicadae*	In vitro Human MHCC97H hepatocellular carcinoma cells	2-DE followed by MALDI-TOF/TOF MS	Western blotting	Wang, H., et al., 2014
*Pleurotus sajor-caju*	n-hexane, chloroform, ethyl acetate, ethanol and ethanol/water *Pleurotus sajor-caju* fruiting body extract	In vitro Human colorectal adenocarcinoma HCT-116*^wt^*, *^-Bax^*, *^-p21^* cells	Protein array	Western blotting	Finimundy, T.C., et al., 2018
*Pleurotus eryngii*	PEP 1b, a novel immunoregulatory protein	Raw 265.7 macrophage cells	*iTRAQ labeling followed by LC-MS/MS and bioinformatic analysis	Western blotting	Ma, N., et al., 2020
*Trametes versicolor*	Polysaccharopeptide (PSP)	Ex vivo Human T lymphocytes	2-DE followed by MALDI-TOF MS	Western blotting	Lee, C.L., et al., 2007
*Hericium erinaceus*	Erinacine A	In vitro Human MKN28 and TSGH9201 human gastric carcinoma	2-DE followed by MALDI-TOF/TOF	Western blotting	Kuo, H.C., et al., 2017
*Hericium erinaceus*	Erinacine A	In vitro (DLD-1 and HCT-116) and in vivo (HCT-116 xenograft) human colorectal carcinoma cells	2-DE followed by MALDI-TOF/TOF	Western blotting	Lee, K.C., et al., 2017
*Phellinus linteus*	Proteoglycan P1	In vitro and in vivo (xenograft) HepG2 human hepatocellular carcinoma cells	2-DE followed by MALDI-TOF/TOF	RT-PCR and Western blotting	Li, Y.G., et al., 2013
*Grifola frondosa*	Glycoprotein GFG-3a	In vitro Human gastric cancer cell line SGC-7901	2-DE followed by MALDI-TOF-MS	qRT-PCR and Western blotting	Cui, F., et al., 2016
*Antrodia cinnamomea*	*Antrodia cinnamomea* fruiting body ethanolic extract	In vitro Liver cancer (HepG2 and C3A) and normal liver (Chang’s) cell lines	2D-DIGE followed by MALDI-TOF MS and bioinformatic analysis	Immunoblot analysis	Chen, J.F., et al., 2020
*Auricularia auricula*	Three types of *Auricularia auricula* isolates (FD, BS, BT)	In vitro hepatocellular cancer cell lines (HCT-15, huh-7, SK-MEL-5, SNU-213, and SNU-484)	2-DE-GE followed by MALDI-TOF-MS	RT-PCT and Western blotting	Kang, M.A., et al., 2020
*Sporisorium reilianum*	*Sporisorium reilianum* polysaccharide WM-NP-60	In vitro Human colorectal adenocarcinoma HCT-116 cells	Tandem mass tag (TMT) labeling followed by nano-HPLC-MS/MS and bioinformatic analysis	qRT-PCR and Western blotting	Kan, L., et al., 2020
*Agrocybe aegerita*	Fraction 2 proteins of *Agrocybe aegerita* (AA-f2-MNC-CM)	In vitro Human leukemic U937 cells	2-DE followed by MALDI-TOF-MS	No	Wang, Y.T., et al., 2004
*Nectria haematococca*	Fungal immunomodulatory protein (FIP) from *Nectria hematococca* (FIP-nha)	Human lung adenocarcinoma (A549 and NCI-H2347) calls in vitro and A549 in vivo	iTRAQ (isobaric tag for relative and absolute quantitation) followed by quadrupole-orbitrap MS and bioinformatic analysis	Western blotting	Xie, Y., et al., 2018
*Agaricus bisporus*	ABL-*Agaricus bisporus* lectin conjugated with CaCO_3_ nanoparticles (ABL- CaCO_3_NPs)	In vitro Human breast cancer cells (MCF-7)	2-DE followed by LC-MS and bioinformatic analysis	No	Mahmood, R.I., et al., 2021
*NA	Ergosterol	In vitro Murine macrophage cell line RAW 264.7	2-DE followed by MALDI-TOF/TOF-MS	Western blotting	Kuo, C., et al., 2011
*Lentinus edodes*, *Ganoderma lucidum*, *Agaricus brasiliensis* (=*blazei* ss. Heinem.), *Grifola frondosa*, *Pleurotus ostreatus*, and *Trametes versicolor* combined in a commercial medicinal mushroom extract blend Agarikon.1	Various	In vivo Murine colorectal carcinoma CT26.WT model	Tandem mass tag (TMT) labeling followed by LC-MS/MS and bioinformatic analysis	Western blotting	Jakopovic, B., et al., 2020

*NA: not applicable; *iTRAQ: isobaric tag for relative and absolute quantitation.
